# Counterfactual Desirability

**DOI:** 10.1093/bjps/axv023

**Published:** 2015-08-04

**Authors:** Richard Bradley, H. Orri Stefánsson

**Affiliations:** 1 Department of Philosophy, Logic and Scientific Method London School of Economics and Political Science Houghton Street London WC2A 2AE, UK; 2 Fondation Maison des Sciences de l’Homme Collége d’études mondiales 190 avenue de France Paris 75013 France France hlynur.orri@gmail.com

## Abstract

The desirability of what actually occurs is often influenced by what could have been. Preferences based on such value dependencies between actual and counterfactual outcomes generate a class of problems for orthodox decision theory, the best-known perhaps being the so-called Allais paradox. In this article we solve these problems by extending Richard Jeffrey’s decision theory to counterfactual prospects, using a multidimensional possible-world semantics for conditionals, and showing that preferences that are sensitive to counterfactual considerations can still be desirability-maximizing. We end the article by investigating the conditions necessary and sufficient for a desirability function to be a standard expected-utility function. It turns out that the additional conditions imply highly implausible epistemic principles.

1 Two Paradoxes of Rational Choice
2 Jeffrey Desirability
3 Counterfactuals
  3.1 Probability and desirability of counterfactuals
  3.2 Representations
4 Counterfactual-Dependent Preferences
  4.1 Preference actualism and desirability maximization
  4.2 Modelling Allais's and Diamond’s preferences
5 Ethical Actualism and Separability
  5.1 Independence and additive separability
  5.2 Ethical actualism
  5.3 Expected utility, separability, and ethical actualism
6 Concluding Remarks
7 Appendix

The desirability of what actually occurs is often influenced by what could have been. Suppose you have been offered two jobs, one very exciting but with a substantial risk of unemployment, the other less exciting but more secure. If you choose the more risky option and as a result become unemployed, you might find that the fact that you could have chosen the risk-free alternative makes being unemployed even worse. In addition to experiencing the normal pains of being out of job, you might then be filled with regret for not having chosen the risk-free alternative. On other occasions something different from regret explains the dependence of our assessments of what is the case on what could have been. Suppose a patient has died because a hospital gave the single kidney that it had available to another patient. Suppose also that the two patients were in equal need of the kidney, had equal rights to treatment, and so on Now if we were to learn that a fair lottery was used to determine which patient was to receive the kidney, then most of us would find that this makes the situation less undesirable than had the kidney simply been given to one of them. For that at least means that the patient who died for lack of a kidney had had a chance to acquire it. In other words, had some random event turned out differently than it actually did, the dead patient would have lived.

This desirabilistic dependency between what is and what could have been creates well-known problems for the traditional theory of rational choice under risk and uncertainty, as formulated by John von Neumann and Oskar Morgenstern ([2007]) and Leonard Savage ([1972]). The first example is just a simplified version of Maurice Allais's ([1953]) infamous paradox, whereas the latter is an instance of a decision theoretic problem identified decades ago by Peter Diamond ([1967]). In this article we use a framework based on a combination of Richard Jeffrey’s ([1990]) decision theory and a multidimensional possible-world semantics for counterfactual conditionals (Bradley [2012]) to explore the above dependency.

Section 1 explains the two paradoxes and why they cast doubt on a rationality postulate, known as ‘separability’. Separability is assumed by a class of mainstream decision theories—for which we will reserve the label ‘expected utility theory’ (EU theory for short)—including those of von Neumann and Morgenstern (where it is called ‘independence’) and Savage (where it is called the ‘sure-thing’ principle). Separability is not presupposed by Richard Jeffrey’s decision theory, however. His is a theory of desirability maximization that is not an EU theory (in the vocabulary adopted in this article). This makes his theory a good candidate for handling Allais's and Diamond’s examples but, as we explain in Section 2, the lack of counterfactual prospects in his theory means that it too cannot easily represent the preferences revealed in these examples. To overcome this problem, in Section 3 we introduce counterfactuals into Jeffrey’s theory and then, in Section 4, show how this makes it possible to represent such preferences as maximizing the value of a Jeffrey desirability function, even though they cannot be represented as maximizing expected utility. In Section 5 we show that, contrary to what decision theorists and philosophers have typically assumed, a second assumption of ‘ethical actualism’, quite different from the aforementioned separability property, is also involved in the clash between Allais's and Diamond’s preferences and EU theory. Indeed it turns out that ethical actualism and separability are both necessary for expected-utility maximization and, given the other assumptions of Jeffrey’s theory, sufficient for it. Since ethical actualism and separability impose unreasonable constraints on agents’ attitudes, we conclude that rationality does not require that agents maximize expected utility.

## 1 Two Paradoxes of Rational Choice

The Allais paradox has generated a great deal of discussion amongst philosophers, psychologists, and behavioural economists. The paradox is generated by offering people a pair of choices between different lotteries, each of which consists in tickets being randomly drawn. First people are offered a choice between a lottery that is certain to result in the decision-maker receiving a particular prize, say £2400, and a lottery that could result in the decision-maker receiving nothing, but could also result in the decision-maker receiving either as much as or more than £2400. The situation can be represented as a choice between the lotteries *L*_1_ and *L*_2_ below, where, for instance, *L*_1_ results in the decision-maker receiving a prize of £2500 if one of tickets number 2 to 34 is drawn:


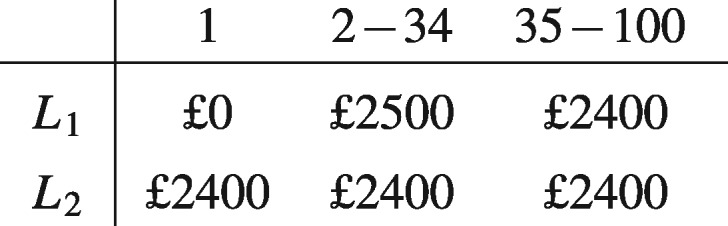


Having made a choice between *L*_1_ and *L*_2_, people are asked to make a second one, this time between lotteries *L*_3_ and *L*_4_:


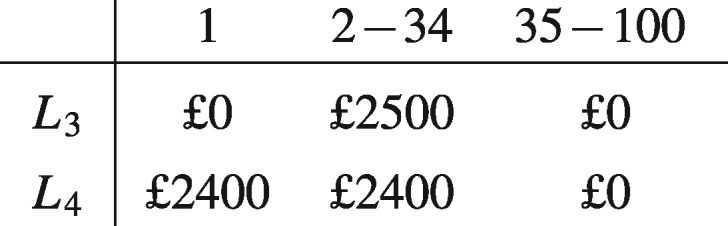


Repeated (formal and informal) experiments have confirmed that people tend to choose and strictly prefer *L*_2_ over *L*_1_ and *L*_3_ over *L*_4_. (See (Kahneman and Tversky [1979]) for discussion of an early experiment of the Allais paradox.) One common way to rationalize this preference, which we will refer to as ‘Allais's preference’, is that when choosing between *L*_1_ and *L*_2_, the possibility of ending up with nothing when you could have received £2400 for sure outweighs the possible extra gain of choosing the riskier alternative, since receiving nothing when you could have gotten £2400 for sure is bound to cause considerable regret (see, for example, Loomes and Sugden [1982] and Broome [1991]). When it comes to choosing between *L*_3_ and *L*_4_, however, the desire to avoid regret does not play as strong role, since decision-makers reason that if they choose *L*_3_ and end up with nothing then they would, in all likelihood, have received nothing even if they had chosen the less risky option *L*_4_.

Intuitively rational as it seems, Allais's preference is inconsistent with the most common formal theory of rational choice: EU theory (assuming, that is, that the probabilities of each ticket are the same in the two choice situations.) According to EU theory, all rational preferences over prospects can be represented as maximizing the expectation of a utility function. Formally, let any prospect or option *f* be a function from a set of states of the world, $S=\{S_{i}\}$, to a set of consequences, with $f(S_{i})$ being the consequence of exercising option *f* when the state of the world is *S_i_*. The expected utility of a prospect *f* is then defined by^1^:

$$EU(f)=\sum_{S_{i}\varepsilon \text{S}}u(f(S_{i})).\mathrm{Pr}(S_{i}),$$

where Pr is a probability measure on the states and *u* a utility measure on consequences. In von Neumann and Morgenstern’s theory, the probabilities on states are objective and the prospects are called lotteries; in Savage’s more general framework, the probabilities are subjective and the prospects called acts. But these differences will not matter to our discussion.

In the usual manner let ≿ represents the agent’s ‘… is least as preferred as …’ relation between alternatives and ≻ and ∼ the corresponding strict preference and indifference relations between them. Then EU theory states that for any rational agent:

(1) f≻g  if and only if  EU(f)>EU(g).

When this holds for someone’s preferences, we say that the *EU* function represents their preferences.

The problem that the Allais paradox poses to decision theory is that there is no way to represent Allais's preference over lotteries in terms of the maximization of the value of a function with the EU form. To see this, let us assume that in both choice situations the decision-maker considers the probability of each ticket being drawn to be 1/100. Then if Allais's evaluation of the alternatives is in accordance with the EU equation, Allais's preference implies:

(2) u(£0)+(33u(£2500))+(66u(£2400))<100u(£2400),

and

u(£2400)+33u(£2400)<u(£0)+33u(£2500).

But the latter implies:

$$\begin{array}{l} \;u(£2400)+33u(£2400)+66u(£2400)=100u(£2400)\\ <u(£0)+33u(£2500)+66u(£2400), \end{array}$$

in contradiction with the inequality in Equation 2. Hence, there is no EU function that simultaneously satisfies $EU(L_{1})<EU(L_{2})$ and $EU(L_{4})<EU(L_{3})$. In other words, there is no way to represent a person who (strictly) prefers $L_{2}$ over *L*_1_ and *L*_3_ over *L*_4_ as maximizing utility as measured by the EU function. Since all rational preference should, according to EU theory, be representable as maximizing expected utility, this suggests that either Allais's preference is irrational or EU theory is incorrect. Hence the ‘paradox’: many people both want to say that Allais's preference is rational and both that EU theory is the correct theory of practical rationality.

Another way to see that Allais's preference cannot be represented as maximizing the value of an EU function is to notice that the preference violates a separability condition on preferences that is required for it to be possible to represent preferences by an EU function. The condition requires that when comparing two alternatives whose consequences depend on what state is actual, rational agents only consider the state(s) of world where the two alternatives differ. More formally:


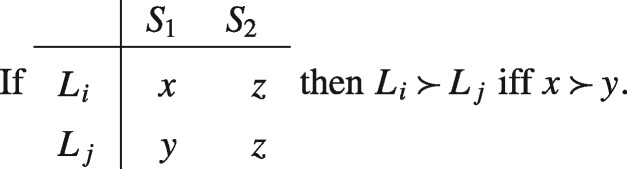


In the choice problem under discussion, this means that you only need to consider the tickets that give different outcomes depending on which alternative is chosen. Hence, you can ignore the fourth column—tickets 35–100—both when choosing between *L*_1_ and *L*_2_ and when choosing between *L*_3_ and *L*_4_, since these tickets give the same outcome no matter which alternative is chosen. When we ignore this column, however, alternative *L*_1_ becomes identical to *L*_3_, and *L*_2_ to *L*_4_. Hence, by simultaneously preferring *L*_2_ over *L*_1_ and *L*_3_ over $L_{4}$, the decision maker seems to have revealed an inconsistency in her preferences.

The second example discussed in the introduction generates a paradox similar to Allais's if we assume that there is nothing irrational about strictly preferring a lottery that gives the patients an equal chance of receiving the kidney over giving the kidney to either patient without any such lottery being used. If we call the patients Ann and Bob, and let *ANN* represent the outcome where Ann receives the kidney and *BOB* the outcome where Bob receives the kidney. To represent the aforementioned attitude—which we will refer to as ‘Diamond’s preference’—as maximizing the value of an EU function, it has to be possible to simultaneously satisfy:

$$\begin{array}{l} u(ANN)<0.5u(ANN)+0.5u(BOB),\\ u(BOB)<0.5u(ANN)+0.5u(BOB). \end{array}$$

But that is of course impossible: an average of the values *u*(*ANN*) and u(BOB) can never be greater than both values *u*(*ANN*) and *u*(*BOB*).

Again, we can see the tension between Diamond’s preference and standard theories of rational choice by noticing that it violates separability. An implication of separability is that, given the prospects displayed below, where *E* represents the outcome of some random event (for example, a coin toss), $L≻L_{A}$ if and only if $L_{B}≻L_{A}$ and $L≻L_{B}$ if and only if $L_{A}≻L_{B}$. Hence, Diamond’s preference in conjunction with separability implies a contradiction.


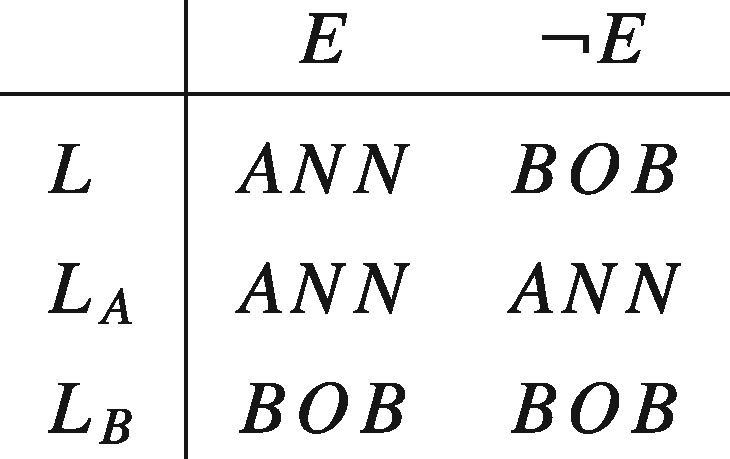


The fact that both Allais's and Diamond’s preferences involve a violation of separability and that their preferences seem intuitively rational (or at least not irrational), casts doubt on separability as a rationality postulate. Moreover, both the desire to avoid regret, as manifested in Allais's preference, and the concern for giving each patient a ‘fair chance’, which seems to be what underlies Diamond’s preference, have something to do with counterfactuals. Regret, at least in the situation under discussion, is a bad feeling associated with knowing that one could have acted differently and that if one had things would have been better. And to say that even if Bob did not receive a kidney, he nevertheless had a chance seems to mean that there is a meaningful sense in which things could have turned out differently—for instance, a coin could have come up differently—and if they had, Bob would have received the kidney. So both Allais and Diamond violate the formal separability requirement of standard decision theories since they judge that the value of what actually occurs at least partly depends on what could have been, that is, on counterfactual possibilities.^2^

Perhaps for the reason discussed above, some economists and philosophers have thought that separability as a requirement on preference is implied by an evaluative assumption we call ‘ethical actualism’. Informally put, ethical actualism is the assumption that only the actual world matters, so that the desirability of combinations of what actually occurs and what could have occurred only depends on the desirability of what actually occurs. In a well-known defence of separability, Nobel Laureate Paul Samuelson argues that it would be irrational to violate ethical actualism, and since he thinks that ethical actualism implies separability, he takes this argument to show that it would be irrational to violate separability. The separability postulate Samuelson was defending, which is implied by what we above called separability, states that if some outcome ${\left(A\right)}_{1}$ is at least as good as ${\left(B\right)}_{1}$ and ${\left(A\right)}_{2}$ is at least as good as ${\left(B\right)}_{2}$, then an alternative that results in ${\left(A\right)}_{1}$ if a fair coin comes up heads but ${\left(A\right)}_{2}$ if it comes up tails is at least as good as an alternative that results in ${\left(B\right)}_{1}$ if the coin comes up heads but ${\left(B\right)}_{2}$ if it comes up tails. Here is Samuelson’s informal justification of the axiom:

[…] either heads or tails must come up: if one comes up, the other cannot; so there is no reason why the choice between ${\left(A\right)}_{1}$ and ${\left(B\right)}_{1}$ should be ‘contaminated’ by the choice between ${\left(A\right)}_{2}$ and ${\left(B\right)}_{2}$. (Samuelson [1952], pp. 672–3)

In other words, the reason an evaluation or ordering of alternatives should satisfy separability is that there should be no desirabilistic dependencies between mutually incompatible outcomes; or, our preferences should satisfy separability since our evaluation of outcomes should satisfy ethical actualism.

Some philosophers and decision theorists have cited Samuelson’s remark favourably. John Broome, who takes it to at least provide a ‘*prima facie* presumption in favour of [separability]’, rhetorically asks: ‘How can something that never happens possibly affect the value of something that does happen?’ (Broome [1991], p. 96). But however closely related ethical actualism and separability might seem to be, the former does not (by itself) imply the latter. In fact the two are based on different, though consistent, intuitions. The former expresses the idea that only what actually happens matters, while the latter expresses the idea that the desirability of what would be the case if one set of conditions held true is independent of what would be the case if some other set of conditions did. To see that these are different requirements consider the set of prospects displayed in the matrix below.


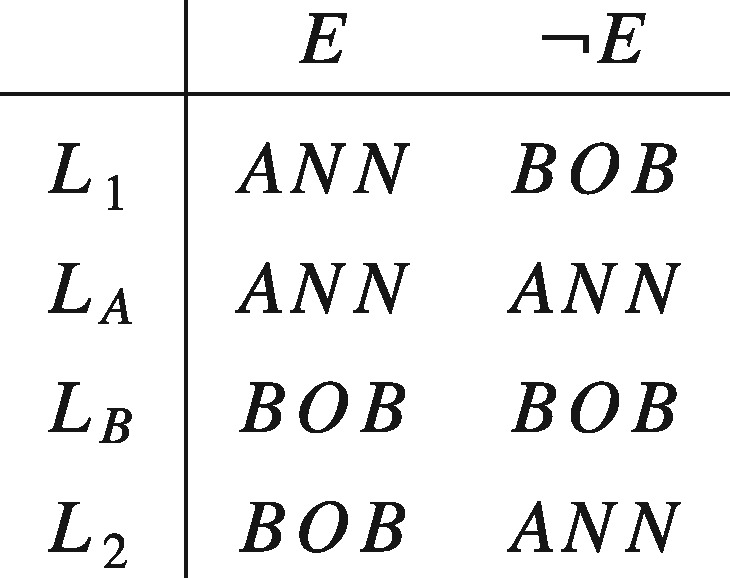


Now, as we have seen, separability requires that $L_{1}≻L_{A}$ if and only if $L_{B}≻L_{2}$. On the other hand, ethical actualism requires that, conditional on *E* being true, $L_{1}∼L_{A}$ and $L_{B}∼L_{2}$. Clearly, in the absence of further restrictions, it is possible for one of these to hold without the other. So even if Samuelson and Broome are right about the intuitive appeal of ethical actualism, this does not establish that separability is rationally required.

## 2 Jeffrey Desirability

Not all decision theories assume separability. In particular, the version of decision theory developed by Richard Jeffrey ([1990]) makes do with much weaker rationality conditions on preference. Indeed, although in an informal sense it is true that Jeffrey’s theory prescribes choosing actions that have the best expected consequences, the value function that rational agents maximize on his theory is, strictly speaking, a desirability function but not an expected-utility function (the difference is explained below). The question that we now want to explore is whether we can represent Allais's and Diamond’s preferences as maximizing Jeffrey desirability, even though they cannot be represented as expected-utility maximizing.^3^

In Jeffrey’s theory preferences are numerically represented by a desirability function, *Des*, and a corresponding probability measure, *Prob*, both defined on a Boolean algebra of propositions—that is, a set of propositions closed under negation, conjunction, and disjunction—from which the impossible proposition has been removed. If we take a proposition to be a set of possible worlds, we can state his theory more formally as follows: Let W be the universal set of possible worlds and Ω the set of subsets of W (that is, the power set of W). Then desirability and probability measures are defined over Ω, elements of which (the propositions) we denote by non-italic uppercase letters (A, B, C, and so on). We can thus think of each way in which proposition A can be true as a world that is compatible with the truth of A. Assuming for simplicity that there are at most countably many mutually exclusive worlds compatible with A, then the Jeffery desirability of a proposition is given by:

$$Des(\text{A})=\sum_{w_{i}\in \text{W}}Des(\{w_{i}\}).Prob(\{w_{i}\}|\text{A}).$$

One way to think of a desirability measure is as an extension of the utility measure on consequences that EU theory postulates (that is, on possible worlds or maximally specific propositions) to the entire Boolean algebra of prospects formed from them.^4^ For given such a utility measure on consequences/worlds, we can define the desirability of any prospect as the conditional expectation of utility, given the truth of the prospect. Note that if for each *w_i_* such that $Prob(\{w_{i}\}|\text{A})>0$, we can find a proposition *S_i_* that is probabilistically independent of A and such that *w_i_* is the consequence of A in *S_i_*, then it will be the case that $Prob(\{w_{i}\}|\text{A})=Prob({\text{S}}_{i})$ and the desirability of A will be its unconditional expectation of utility relative to the probability distribution over the *S_i_*. But this is a special case and in general desirabilities may not take this form.

Our interest in Jeffrey’s theory lies mainly in the possibility that Allais's and Diamond’s preferences are desirability-maximizing, but there is a second reason for favouring it over the expected utility theories of Savage and others. To apply Savage’s theory one must model the decision problem in a very specific way. In particular, one must find states of the world that are probabilistically independent of the acts amongst which one may choose and consequences whose utilities are independent of the states of the world in which they are realized. In effect, this latter requirement means that consequences must be identified by propositions that are maximally specific about everything that matters to the agent. Real agents are rarely able to formulate decision problems in a manner that meets these requirements. But if they do not, then there is no guarantee that by maximizing expected utility relative to the coarse-grained specification of the decision problem (that is, relative to the ‘small-world’ decision problem) then they do so relative to a fully refined description of it (that is, relative to the ‘grand-world’ problem).^5^ In contrast, Jeffrey’s notion of desirability is partition-invariant in the sense that if a proposition, A, can be expressed as the disjoint disjunction of both $\left\{{\text{B}}_{1},{\text{B}}_{2},{\text{B}}_{3}...\right\}$ and $\left\{{\text{C}}_{1},{\text{C}}_{2},{\text{C}}_{3}...\right\}$, then^6^$\sum_{{\text{B}}_{i}\in \text{A}}Prob({\text{B}}_{i}|\text{A}).Des({\text{B}}_{i})=\sum_{{\text{C}}_{i}\in \text{A}}Prob({\text{C}}_{i}|\text{A}).Des({\text{C}}_{i}).$It follows that applying the rule of desirability maximization will always lead to the same recommendation, irrespective of how the decision problem is framed, while EU theory may recommend different courses of action, depending on how the decision problem is formulated.

In Jeffrey’s theory acts are just propositions that can be made true at will, and so the desirabilities of acts will partly depend on the conditional probabilities of their consequences, given the performance of the acts. As a result, separability can fail. For instance, consider two acts, *A* and *B*, with consequences contingent on states *S*_1_ and *S*_2_, as displayed below:


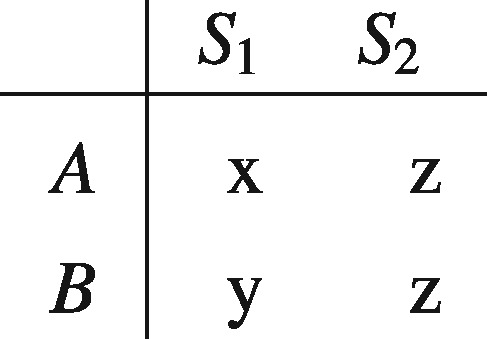


Separability requires that A≻B if and only if x ≻ y. But if z is considered a more desirable outcome than both x and y, and *A* makes *S*_2_ more likely than does *B*, then *A* might be assigned a higher Jeffrey desirability than *B*, even when x is not preferred to y. So Jeffrey’s theory does not require separability.

Unfortunately, this does not completely solve our problem of making Allais's and Diamond’s preferences consistent with decision theory. For although Jeffrey’s theory does not imply separability, the theory as it is usually applied is also inconsistent with Allais's and Diamond’s preferences. Let us focus on the Diamond paradox to see the problem. ${\text{L}}_{B}$ now represents the set of worlds where Bob gets the kidney no matter what, ${\text{L}}_{\neg \text{B}}$ the set of worlds where Ann gets the kidney no matter what, and L the set of worlds where the toss of a fair coin decides who gets the kidney. Then for Diamond’s preference to be compatible with Jeffrey’s theory, it would seem that there has to be a function, *Des*, such that:^7^

$$\begin{array}{l} Des(ANN)<Des(ANN).Prob(ANN|\text{L})+Des(BOB).Prob(BOB|\text{L}),\\ Des(BOB)<Des(ANN).Prob(ANN|\text{L})+Des(BOB).Prob(BOB|\text{L}). \end{array}$$

But again, a probability mixture of the desirabilities of *ANN* and *BOB* can never exceed the desirability of both *ANN* and *BOB*.

What this shows is that there is more at play than just the failure of separability in the explanation of Allais's and Diamond’s preferences. For the standard representation of the two problems, and our application of Jeffrey’s theory to them, implicitly builds in the aforementioned assumption of ethical actualism. Without this assumption (but still assuming that the desirability of Ann or Bob getting the kidney is independent of the random event E), Jeffrey’s theory just says:

Des(L)=Des(ANN∧L).Prob(ANN|L)+Des(BOB∧L).Prob(BOB|L)

and nothing requires that Des(ANN∧L)=Des(ANN) or *Des*(*BOB∧L*) =Des(BOB).

It seems then that the way to accommodate the Allais's and Diamond’s preferences within Jeffrey’s framework is just to specify the consequences of actions sufficiently broadly so as to make it intelligible that, for instance, Ann getting the kidney in a fair lottery is a different consequence from her getting it as a part of a process that made it certain she would receive it. More generally, the notion of consequence should be broadened to take account of what could have happened as well as what did happen. Just such a response to the two paradox has been suggested by, for instance, John Broome ([1991]), who argues that if regret and fairness matter to an agent then that should be part of the description of the outcomes of lotteries,^8^ and by Paul Weirich ([1986]), who argues that the correct way to account for the risk attitudes displayed in the Allais paradox is to allow that the risk involved in exercising an option counts as one of its consequences.

Solutions of this kind will be unsatisfactory, however, if they involve introducing new primitive consequences in the representation of the decision problem without explaining their relationship to the available actions. In particular, they must explain what it is about the form of the lottery L that makes Des(ANN∧L)>Des(ANN). It is not, in our view, sufficient to say that the first outcome is fair while the latter is not; what is needed is an explanation of why the first outcome is fair. Moreover, to avoid trivializing decision theory by making it allow that any possible choice can be rational, we should require that exercises of this kind–where new propositions (or consequences) are created to make seemingly problematic preferences compatible with decision theory–adhere to some independently plausible principles as Broome himself points out (see Broome [1999]; see also discussion of this in Stefánsson [forthcoming]).

In the context of Jeffrey’s framework, avoiding these objections requires a specification of the propositional structure of lotteries and acts, and the attitudes that they support. We do so by widening the domain of Jeffrey’s theory to include counterfactual propositions and showing that the properties that generate Allais's and Diamond’s paradoxes–respectively, regret and fairness–then emerge as a relationship between factual and counterfactual propositions. Our solution thus provides at least a partial explanation of the preferences that generate these paradoxes, by highlighting the effects counterfactuals have on the desirabilities of the prospects in question.^9^ Moreover, our solution does not trivialize decision theory, since the domain of Jeffrey’s original theory is extended in a principled way (to be explained in the next section) and the resulting theory requires that people’s preferences between all propositions satisfy the so-called Bolker-Jeffrey axioms (which we introduce in Section 3.2).

This solution to the problems raised by Allais and Diamond is not ad hoc, we think, since decision theory should, independently of these problems, allow for the value dependencies one often finds between actual and counterfactual outcomes. And this solution has the advantage over the refinement solution suggested by Broome, that whereas he solves each of the two problems under discussion by introducing different properties to the description of the outcomes, our solution solves both problems at once by introducing counterfactual conditionals to the domain of Jeffrey’s decision theory. Hence, while the typical refinement solution to the problems raised by Allais and Diamond treats the two preferences as having nothing in common except violation of separability, our solution makes explicit that these are two instances of a general type of preference that causes trouble for EU theory, namely, counterfactual-dependent preference.

Before introducing counterfactual conditionals to Jeffrey’s theory, let us first briefly explain why introducing indicative conditionals to Jeffrey’s theory (as done, for example, in Bradley [1998], [2007]) will not solve the problem of representing Allais's and Diamond’s preferences. An indicative conditional is generally considered to be what Jonathan Bennett calls ‘zero intolerant […] meaning that such a conditional is useless to someone who is really sure that its antecedent is false’ (Bennett [2003], p. 45). In other words, if ‘↦’ represents the indicative conditional connective, then A↦B is informative for someone who thinks that A might be true (where ‘might’ is understood epistemically, not merely logically or metaphysically). But A↦B provides no information about a world where one is certain that A is false. (Hence, its ‘uselessness’ to someone who is certain that A is false.^10^) It is therefore plausible to assume, as Bradley does, that Des(¬A∧(A↦B))=Des(¬A), since if A is believed to be false, then A ↦B makes no desirabilistic difference. Thus the conditionals that generate the paradoxes under discussion cannot be indicative conditionals, since the problems they generate consist exactly in the fact that they have desirabilistic impact when their antecedents are believed to be false.

What we need to do therefore is introduce counterfactual conditionals into Jeffrey’s theory. Jeffrey himself recognized the need to do so and tried to solve the problem of providing an account of counterfactuals, but in his own view did not succeed.

(If I had, you would have heard of it. There’s a counterfactual for you.) In fact, the problem hasn’t been solved to this day. I expect it’s unsolvable. (Jeffrey [1991], p. 161)

Jeffrey was unduly pessimistic. Since he made this remark there has been considerable progress in the understanding of counterfactuals, progress that we now build on.

## 3 Counterfactuals

Our problem is to find a way of representing counterfactual propositions (counterfactuals for short) in a way that enables us to exploit the resources of Jeffrey’s decision theory to model Allais's and Diamond’s preferences. To do so we extend standard possible world modelling of propositions in a natural way by introducing the notion of a possible counteractual world under a supposition. A possible world is a way things might be or might have been. A possible counteractual world under the supposition that some *A* is true, on the other hand, is just a way things might be, or might have been, were *A* true.

If world *w_A_* could be the case under the supposition that *A*, then we will say that *w_A_* is a possible counteractual *A*-world. If *A* is false, *w_A_* will be said to be strictly counterfactual. (Any counteractual *A*-world is strictly counterfactual relative to any possible world in which *A* is false for instance. But counteractual worlds are not always strictly counterfactual: if *A* is true then *w_A_* may not only be a possible way things are under that supposition that *A*, but the way things actually are.)

Our basic thesis is that possible counteractual worlds make counterfactual claims true in the same way that possible actual worlds make factual claims true. For instance, if *w_A_* is a counteractual *A*-world at which it is true that *B*, then *w_A_* makes it true that if *A* were the case then *B* would be. Thus the counteractual world in which Obama is born in Kenya and goes to school in Nairobi makes it true that had Obama been born in Kenya, he would have gone to school in Nairobi; the counteractual world in which he is born in Kenya but goes to school in Mombasa makes it false.

To illustrate this thesis, consider a simple model based on the set W $=\text{and}\{w_{1},w_{2},w_{3},w_{4},w_{5}\}$ of just five possible worlds (which are the primitives of the model) and the corresponding set Ω of its subsets, including the events A $=\{w_{1},w_{2},w_{3}\}$, Ā $=\{w_{4},w_{5}\}$, B $=\{w_{1},w_{2},w_{4}\}$, and C $=\{w_{1},w_{3},w_{5}\}$, which are the sets of worlds at which it is true that *A*, ¬A, *B*, and *C* (throughout, we use Ā to denote W − A), respectively. Relative to the set of possible worlds W, a supposition induces a set of possible counteractual worlds. The supposition that *A*, for instance, induces the set of counteractual *A*-worlds, ${\text{W}}_{A}=\{w_{1},w_{2},w_{3}\}$, and the corresponding set of sets of counteractual worlds, Ω*_A_*, containing conditional events ${\text{B}}_{A}=\{w_{i}\in {\text{W}}_{A}:w_{i}\in \text{B}\}=\{w_{1},w_{2}\}$, ${\text{C}}_{A}=\{w_{1},w_{3}\}$ and so on. The supposition that *A* is false induces a different set of counteractual worlds—namely, ${\text{W}}_{\bar{\text{A}}}=\{w_{4},w_{5}\}$—and a corresponding set of conditional events, $\Omega_{\bar{\text{A}}}$. The supposition that *B* induces yet another, and And so on. Note that we have adopted the convention of denoting sets of worlds with non-italicized letters, with A denoting the set of worlds at which it is true that *A*, and ${\text{B}}_{A}$ denoting the set of A-worlds at which it is true that *B*. Also note that the same world can represent a potentially actual world and a counteractual world under a supposition: *w*_1_, for instance, can represent the actual world (if A, B, and C are all true), but also the world that would be actual if, say, A were true.

For simplicity, we restrict attention to a single supposition for the moment, namely, the supposition that *A*. The set of elementary possibilities is then given by a subset, Ϝ, of the cross-product of W and ${\text{W}}_{A}$, which can be presented in tabular form as follows:


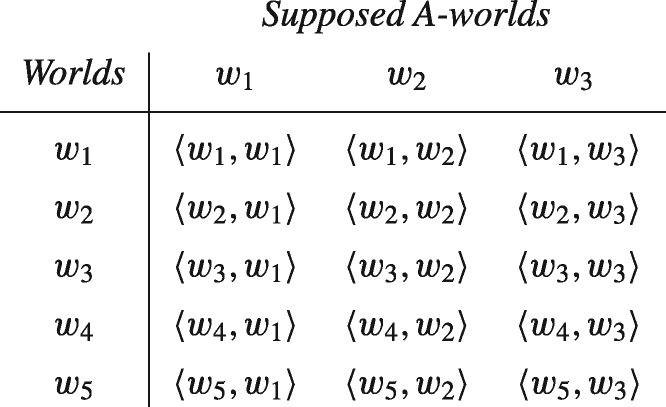


Each ordered pair $\omega_{ij}=\langle w_{i},w_{j}\rangle$ appearing in the cells of the table represents an elementary possibility: that *w_i_* is the actual world and that *w_j_* is the counteractual *A*-world. Sets of such possibilities will serve for us as propositions. Factual propositions are given by unions of rows of the table. The proposition that *A*, for instance, is given by the first, second, and third rows of the table, while that of *B* by the first, second, and fourth. Conditional propositions, on the other hand, are given by unions of columns of the table. The proposition ‘if *A* then *B*’, for instance, is given by the first and second columns of the table, while the proposition ‘if *A* then *C*’ is given by the first and third columns. Conjunctions, disjunctions, and negations of propositions (conditional or otherwise) are given by their intersection, union, and complements.

The above table implicitly assumes that every element of W × ${\text{W}}_{A}$ is a possible combination of facts and counterfacts, but this assumption is easy to dispense with. To generate a space of elementary possibilities, Ϝ, we make use of a selection function on worlds that determines which counteractual worlds are ‘accessible’ from them. Formally, a selection function, *f*, is a mapping from W×Ω to Ω satisfying, for all w∈W and A ⊆ W,

1. f(w, A) ⊆ A;
2. f(w, A) =Ø⇔ A =Ø;
3. If w∈A then w∈f(w, A).

The first condition simply states that counteractual worlds under the supposition that *A* must be worlds at which it is true that *A*, and the second that the set of counteractual worlds is empty only if the supposition is contradictory. The third condition requires that any world at which it is true that *A* must be a possible counteractual *A*-world. This condition is termed ‘weak centring’, in contrast to its stronger ‘cousin’ that is typically assumed in the semantics of counterfactuals:

Centring: If w∈A then f(w, A) = {*w*}.

Centring expresses a particular conception of the relation between factual and counterfactual possibility, according to which what is actually true determines what might have been true under any supposition consistent with the actual truth. This is surely right for epistemic possibility: if an agent takes the actual world to be *w*, and knows that *A* is true at w, then it should not be epistemically possible, according to her, that any world other than *w* is the case on the supposition that *A*. Epistemic possibility would seem to be what is at issue when we reason evidentially using indicative conditionals. On the other hand, it is much more controversial whether centring governs causal possibility, and hence whether it is appropriate to counterfactual reasoning. Both Lewis and Stalnaker assume that it is, perhaps because they take counterfactual and evidential reasoning to coincide when what is being supposed is in fact true. But in the absence of a deterministic relationship between two events, it does not seem obviously right to regard the fact of their co-occurrence to imply that the occurrence of one causally necessitated the other. So it is not clear that the assumption is appropriate for counterfactuals. In any case, we do not need to settle the issue here and will for the sake of generality not assume centring.^11^

We now have all the ingredients in place to state our account of counterfactual possibility. As before, let W be a set of possible worlds, Ω& be a Boolean algebra of subsets of W, and $S=\{{\text{S}}^{i}\}\subseteq \Omega$ be a set of *n* suppositions. The elementary possibilities on this account are *n*-tuples of worlds $\langle w,w_{1},...,w_{n}\rangle$, with w∈ W and each $w_{i}\in$ S*^i^*. Propositions are sets of such *n*-tuples of worlds. More formally, a suppositional algebra is a structure 〈W,Ω,S,f,Ϝ,Γ〉 with *f* a selection function from the set of worlds W and set of suppositions S to sets of worlds, which determines a set, Ϝ, of elementary *n*-tuples of worlds by

$$Ϝ:=\{\omega =\langle w,w_{1},...,w_{n}\rangle :w\in \text{W},w_{i}\in f(w,{\text{S}}^{i})\},$$

and Γ is a Boolean algebra of subsets of Ϝ (the propositions).

For any S${}^{i}\in S$, let Ω*_i_* be the power set of S*^i^*. We adopt the convention of denoting subsets of Ω*_i_* by non-italicized capitals subscripted by *i*. Given X ∈ Ω and ${\text{Y}}_{i}\in$ Ω*_i_*, let 〈X, ${\text{Y}}_{1}$, … , ${\text{Y}}_{n}\rangle$ be the element of Γ that is the proposition that X is the case, that *Y*_1_ is or would be the case, on the supposition that *S*^1^ is or was, … , and that *Y_n_* is or would be, on the supposition that *S^n^*. Each such ordered *n*-tuple is thus a coarse-grained but complex proposition concerning both what is and what could be. When there is no risk of ambiguity we drop ‘empty’ notation and write X for 〈X, ${\text{S}}_{1}$, … , ${\text{S}}_{n}\rangle$, the proposition that *X* is the case; ${\text{Y}}_{i}$ for 〈W, ${\text{S}}_{1}$, … , ${\text{Y}}_{i}$, … , ${\text{S}}_{n}\rangle$, the proposition that if *S^i^* is or were the case, then *Y_i_* is or would be; (X, Y*_i_*) for 〈X, ${\text{S}}_{1}$, … ,${\text{Y}}_{i}$, … ,${\text{S}}_{n}\rangle$; and so on. It follows that (X, ${\text{Y}}_{i})=$ X ∩${\text{Y}}_{i},\;\langle {\text{Y}}_{1}$, … ,${\text{Y}}_{n}\rangle =\cap ({\text{Y}}_{i})$ and so on. A special convention is adopted for the propositions S*^i^* serving as suppositions: we will write (S*^i^*)*_i_* for the proposition that if *S^i^* is or were the case, then *S^i^* is or would be. Note that (S*^i^*)${}_{i}=Ϝ$, since for all w∈W, $f(w,{\text{S}}^{i})\in {\text{S}}^{i}$.

Propositions of the form $\langle {\text{Y}}_{1}$, … ,${\text{Y}}_{n}\rangle$, which specify what will or would be the case under each supposition, are of particular interest to our discussion in virtue of their serving as representations of the actions over which agents have preferences. Consider, for instance, the case described by Diamond, which was previously represented in tabular form:


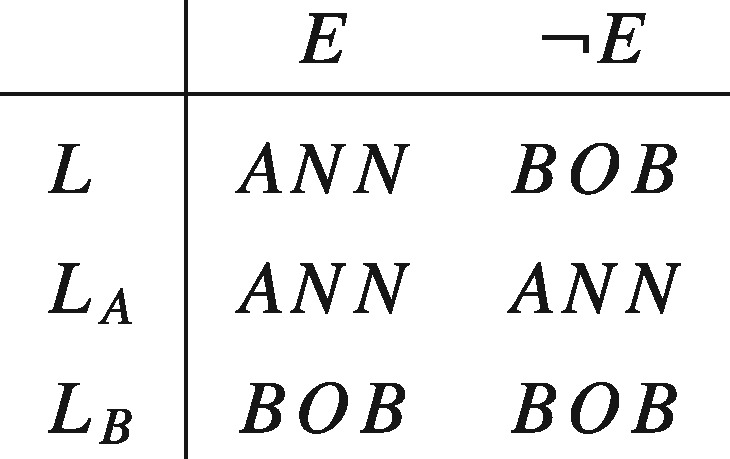


In our framework, *ANN*, *BOB*, and *E*, as well as any Boolean compound of them, would make up the set of factual propositions, with *E* and ¬E serving as the suppositions of interest. The full set of propositions would then be given by the cross product of the set of factual propositions and {E,¬E}, and any Boolean compounds of them. This would contain such conditional propositions as *ANN_E_* —the proposition that Ann would get the kidney if *E* were the case, and $BOB_{\bar{E}}$—the proposition that Bob would get the kidney if *E* were not the case. The lottery *L* would be identified by the complex proposition $\left(ANN_{E},BOB_{\bar{E}}\right)$: a proposition that is a conjunction of the conditional propositions *ANN_E_* and $BOB_{\bar{E}}$, that is, $L=ANN_{E}\cap BOB_{\bar{E}}$. A similar identification occurs for the degenerate lotteries $L_{A}=(ANN_{E},ANN_{\bar{E}})$ and $L_{B}=(BOB_{E},BOB_{\bar{E}})$. Our task now is to say what attitudes one can rationally take to such propositions.

### 3.1 Probability and desirability of counterfactuals

Beliefs about counterfactual possibilities play an important role in our reasoning about what we should do, for they are the means by which we consider the consequences of our actions. Our evaluative attitudes to counterfactual possibilities so too, for instance, the regret we anticipate if we forego opportunities that would have led to desirable outcomes. These attitudes to the counterfacts are at least partially independent of our attitudes to the facts. One might be pretty sure that the match is to be played tomorrow, for instance, but quite unsure as to whether it would be played were it to rain. Equally, one could be quite sure that the match will not be played were it to rain, but quite unsure as to whether it will rain or not. Similarly, our assessment of how desirable something is can differ from our assessment of how desirable it is on the supposition of some condition or other. Even if one prefers to be served a cold beer over a hot chocolate tonight, the preference could be reversed under the supposition that the evening will be very cold.

An agent’s combined uncertainty about what is the case and what would be the case under various possible suppositions will be captured here by a probability mass function, *p*, on the set Ϝ of ordered *n*-tuples of worlds that constitute the elementary possibilities in our model. The mass function *p* measures the joint probabilities of actuality and counteractuality under the various suppositions: $p(\langle w,w_{1},...,w_{n}\rangle )$ is the probability that *w* is the actual world, that *w*_1_ is/would be the counteractual world on the supposition that $S^{1}$, … , and that *w_n_* is/would be the counteractual world on the supposition that *S^n^*. Similarly, we introduce a utility function, *u*, on *n*-tuples of worlds to measure the agent’s evaluations of different combinations of factuality and couterfactuality. For example, $u(\langle w,w_{1},...,w_{n}\rangle )$ will measure the desirability that *w* is the actual world, that *w*_1_ is/would be the counteractual world on the supposition that *S*^1^, … , and that *w_n_* is/would be the counteractual world on the supposition that *S^n^*. For convenience, we assume, as Jeffrey ([1990]) does, that *u* is zero-normalized in the sense that^12^:

$$\sum_{\omega \in Ϝ}u(\omega ).p(\omega )=0.$$

The mass function *p* and utility function *u* induce a corresponding pair of measures, *Prob* and *Des*, on the set Γ of all propositions by means of the following definitions. For all *α*∈Γ (where α could be either factual or conditional)^13^:

$$Prob(\alpha ):=\sum_{\omega \in \alpha }p(\omega ),$$

(3) $$Des(\alpha ):=\sum_{\omega \in \alpha }\frac{u(\omega ).p(\omega )}{Prob(\alpha )}.$$

Within our multidimensional possible world model, *Prob* and *Des* respectively encode the agent’s state of belief and desire regarding both the facts and the counterfacts, with Prob(〈X,${\text{Y}}_{1}$, … ,${\text{Y}}_{n}\rangle )$ measuring the joint probability that *X* is the case and that *Y_i_* is or would be the case if *S^i^*, and Des(〈X,${\text{Y}}_{1}$, … ,${\text{Y}}_{n}\rangle )$ measuring the joint desirability that *X* is the case and that *Y_i_* is or would be the case if *S^i^*.

It is evident that *Prob* satisfies the standard axioms of probability. In virtue of the zero-normalization of *u*, it follows immediately from Equation 3 that *Des* is normalized with respect to the tautology, that is, that Des(Ϝ)=0. Finally, it follows from Equation 3 that *Des* respects Jeffrey’s axiom of desirability.

Desirability: If α∩β=Ø, then
  $$Des(\alpha \cup \beta )=\frac{Des(\alpha ).Prob(\alpha )+Des(\beta ).Prob(\beta )}{Prob(\alpha \cup \beta )}.$$

To see this, let *α* and *β* be two disjoint propositions. Then,

Des(α∪β)=∑ω∈α∨βu(ω).p(ω)Prob(α∪β)=∑ω∈αu(ω).p(ω)Prob(α∪β)+∑ω∈βu(ω).p(ω)Prob(α∪β)=Des(α).Prob(α)+Des(β).Prob(β)Prob(α∪β).

We conclude that our possible world model allows for an extension of Jeffrey’s decision theory to counterfactual propositions.

### 3.2 Representations

We are now in a position to address the question of the conditions under which an agent’s preferences can be represented by a pair of functions, Prob and *Des*, as defined above. In other words, what conditions must her preferences satisfy if they are to be representable in terms of desirability maximization? In fact, most of the work needed to answer this question has already been achieved by showing how to construct a Boolean algebra of counterfactual propositions (indeed, the difficulty in doing so was the main stumbling block in previous attempts to extend Jeffrey’s theory). Given this, we can simply help ourselves to the representation theorem for Jeffrey’s decision theory proved by Ethan Bolker ([1966]) to establish the existence of such a representation.

Bolker imposes two main conditions on preferences in addition to the standard requirement that they be continuous, complete, and transitive. To state them in a form appropriate to our discussion, let ≿ be a complete, transitive, and continuous relation on a Boolean algebra of propositions (construed as sets of *n*-tuples of worlds) and let ≈ and ≻ be the corresponding indifference and strict preference relations on propositions. Then Bolker postulates:

Averaging: If α∩β=Ø, then α≿(α∪β)≿β⇔α≿β.

Impartiality: Suppose α≈β and α∩β=Ø, and that for some γ≈α,β such that α∩γ=β∩γ=Ø, it is the case that α∪γ≈β∪γ. Then for all such *γ*, α∪γ≈β∪γ.

The axiom of averaging is the main rationality constraint on preference required for desirability maximization and was implicitly assumed in our construction of a value function on counterfactual propositions. The essential idea that motivates it is that no proposition can be better (worse) than its best (worst) realization. The proposition that α∪β is consistent with it being the case that *α* and with it being the case that *β*, but not both if *α* and *β* are mutually exclusive. Suppose *α* is preferred to *β*. Then at worst it being the case that α∪β means that *β* and, at best, that *α*. So the desirability one attaches to α∪β should lie between that of *α* and *β*.

Impartiality, on the other hand, is a rationality constraint on the relation between preference and belief. It says that we can test for the equiprobability of any two co-ranked propositions, *α* and *β*, by taking a third proposition *γ*, that is inconsistent with both and checking to see whether α∪γ and β∪γ are ranked together. For suppose that the probability of *α* was in fact greater than that of *β*. Then it would be less likely that *γ*, given that α∪γ, than it would be that *γ*, given that β∪γ. And so α∪γ would be either a less or a more attractive proposition than β∪γ, depending on whether γ≻α,β or α,β≻γ. But if the probabilities of *α* and *β* are the same, then it should be the case for all *γ* inconsistent with both *α* and *β* that α∪γ≈β∪γ.

Let us say that a pair of desirability and probability functions, *Des* and Prob, jointly represent a preference relation ≿ just in case, for all *α* and *β* in the domain of ≿,

α≿β⇔Des(α)≥Des(β).

In this case we say that the pair (*Prob*, *Des*) constitute a Jeffrey representation of the preference relation ≿. What Bolker proved was that, given some technical conditions on the set of propositions (specifically, that they constitute a complete, atomless Boolean algebra) and on the preference relation ≿ (specifically, that it generates a weak and continuous order on the set of propositions), satisfaction of the axioms of averaging and impartiality is necessary and sufficient for the preference relation to be desirability-maximizing. Since the sets of *n*-tuples of worlds forms a Boolean algebra of propositions, his theorem applies directly to our framework. More formally:

**Theorem 1**

Let 〈Γ,⊆〉 be a complete, atomless Boolean algebra of sets of *n*-tuples of worlds (propositions). Let ≿ be a complete, transitive, and continuous relation on Γ−{Ø}. Then there exists a pair of desirability and probability functions, *Des* and *Prob*, respectively on Γ−{Ø} and Γ, that are a Jeffrey representation of ≿ if and only if ≿ satisfies averaging and impartiality (Bolker [1966]).

## 4 Counterfactual-Dependent Preferences

Let us then return to the task of representing Allais's and Diamond’s preferences. Recall that these preferences cannot be represented as maximizing the value of an EU function because the EU equation implies that the value of an outcome in state *S^i^* is desirabilistically independent of any outcome in state *S^j^* that is incompatible with *S^i^*;. This in turn implies that the value of what actually occurs never depends on what merely could have been. (In the next section we define EU functions for suppositional algebras.) But for people with Allais's preference, the desirability of receiving nothing is not independent of whether or not one could have chosen a risk-free alternative. Similarly, for people with preferences like Diamond’s, the desirability of either patient not receiving the kidney is not independent of what would have occurred had some random event turned out differently. So both Allais's and Diamond’s preferences, on this interpretation, are dependent on the truth of counterfactuals. Moreover, the part that causes the violation of EU theory can in both cases be formalized as a relationship between a proposition and a set of worlds that are strictly counteractual.

To make the above claim more precise let’s look at Diamond’s preference first and suppose that Diamond wants to use a coin toss to decide who receives the kidney. Let H be the set of worlds where the coin comes up heads and T the set of worlds where the coin comes up tails (so $\text{T}\equiv \bar{\text{H}}$). Let B be the set of worlds where Bob receives the kidney and A the set of worlds where Ann receives the kidney (so $\text{A}\equiv \text{\&}\bar{\text{B}}$ given the assumption that exactly one of them receives the kidney). We have thus made two simplifying assumptions already. First, it might seem more natural to let H (T) be the set of worlds where the coin comes up heads (tails) if tossed. But nothing is lost, we believe, by this simplification. Second, we have limited our attention to situations where either Ann or Bob receives the kidney. But what is distinctive about Diamond’s preference is what it has to say about situations where a number of individuals have an equal claim on an indivisible good that some, but not all of them get, receive. (Any kind of welfarism, for instance, condemns a situation where none of the patients in need receive the kidney.) Hence, since we want to focus on what is special about this preference, it is justifiable to limit our attention to situations where one of Anna and Bob receives the kidney.

The part of Diamond’s preference that leads to violation of EU theory can then be formulated thus:

(4) $$(\text{H}\cap \text{B},{\text{A}}_{T})≻(\text{H}\cap \text{B},{\text{B}}_{T}).$$

In other words, Diamond prefers the proposition that the coin comes up heads and Bob receives the kidney but Ann would have gotten it had tails come up, to the proposition that the coin comes up heads and Bob receives the kidney and would also have gotten it had the coin come up tails.

Let us then turn to Allais's preference and let R represent the set of worlds where Allais chooses the risky option (which will be *L*_1_ or *L*_3_ depending on the choice situation) and G the set of worlds where Allais is guaranteed to win something. Unlike when representing Diamond’s preference, we need a third (basic) set of worlds to represent Allais's preference, since the worlds where Allais is not guaranteed to win anything are not necessarily the same as the worlds where Allais wins nothing. But it is relative to a situation where Allais has won nothing that the fact that he could have chosen a risk-free alternative makes a difference. Let N denote the set of worlds where Allais wins nothing. Then the preference that causes Allais to violate EU theory can be represented thus:

(5) $$(\text{R}\cap \bar{\text{G}}\cap \text{N},{\bar{\text{G}}}_{\bar{\text{R}}})≻(\text{R}\cap \bar{\text{G}}\cap \text{N},{\text{G}}_{\bar{\text{R}}}).$$

In other words, according to Allais, winning nothing after having made a risky choice is made worse when it is true that had he chosen differently, then he would definitely have won something.

### 4.1 Preference actualism and desirability maximization

We have seen that both Diamond’s and Allais's preferences exhibit a non-trivial sensitivity to counterfactual states of affairs that is manifested in the violation of a condition that we will call ‘preference actualism’ the requirement that preferences for propositions be independent of the strict counterfacts. Formally:

Preference Actualism: For all sets of worlds A, B, C such that $\text{C}\cap \bar{\text{A}}=Ø$:
  $$(\text{C},{\text{B}}_{\bar{\text{A}}})∼(\text{C},{\bar{\text{B}}}_{\bar{\text{A}}}).$$

Preference actualism is of course just a version of the doctrine of ethical actualism that was informally introduced earlier. As we mentioned then, and will explain more precisely in Section 5, it is not sufficient that preferences are separable for them to satisfy preference actualism. An agent may regard the desirability of the counterfacts to be independent without thinking that the counterfacts do not matter. In the Diamond example, such an agent might have preferences $L_{B}≻L≻L_{A}$ in accordance with separability but, contrary to preference actualism, not be indifferent between *L* and *L_A_*, conditional on *E* being the case, perhaps because she values the two relevant strict counterfacts—that Bob or Ann would have got the kidney if *E* had not been the case—differently but positively.

In the Appendix, we prove (as Theorem 15) that preferences that violate preference actualism cannot be represented as maximizing expected utility (as defined in the next section). Since a preference might violate preference actualism without violating separability, this result does not simply follow from the fact that separability is a necessary condition for expected-utility maximization. The independence of these two assumptions has not been recognized in the decision theoretic literature, perhaps because, together with certain assumptions that are either implicitly or explicitly part of standard formulations of EU theory and which do seem to be satisfied in Allais's and Diamond’s examples (in particular, centring and an assumption about the probabilistic independence of counterfacts under disjoint suppositions), preference actualism does imply separability. Indeed, given these assumptions, Allais's and Diamond’s violation of preference actualism can be seen as explaining why they violate separability.

While expected-utility maximization requires adherence to ethical actualism, it is perfectly possible for preferences to satisfy Bolker’s axioms but violate preference actualism. To show this we work again with our simple model based on the set W $=\{w_{1},w_{2},w_{3},w_{4},w_{5}\}$ of five possible worlds and the corresponding set Ω of its subsets, including the events A $=\{w_{1},w_{2},w_{3}\}$, Ā $=\{w_{4},w_{5}\}$, B $=\{w_{1},w_{2},w_{4}\}$, and $\bar{\text{B}}$$=\{w_{3},w_{5}\}$. For present purposes we only need to focus on one supposition, namely, that *A* is false. Then the set of elementary possibilities is given by $W=\{w_{1},w_{2},w_{3},w_{4},w_{5}\}\times \{w_{4},w_{5}\}$ and, in particular, (A∩B, ${\bar{\text{B}}}_{\bar{\text{A}}})=\{\langle w_{1},w_{5}\rangle ,\langle w_{2},w_{5}\rangle \}$ and (A∩B, ${\text{B}}_{\bar{\text{A}}})=\{\langle w_{1},w_{4}\rangle ,\langle w_{2},w_{4}\rangle \}$.

To induce the preferences required, we define a pair of probability and utility mass functions, *p* and *u*, on this set of world pairs, by setting $p(\langle w_{4},w_{5}\rangle )=p(\langle w_{5},w_{4}\rangle )=0$ and assigning the values to remaining possibilities displayed in the following table:


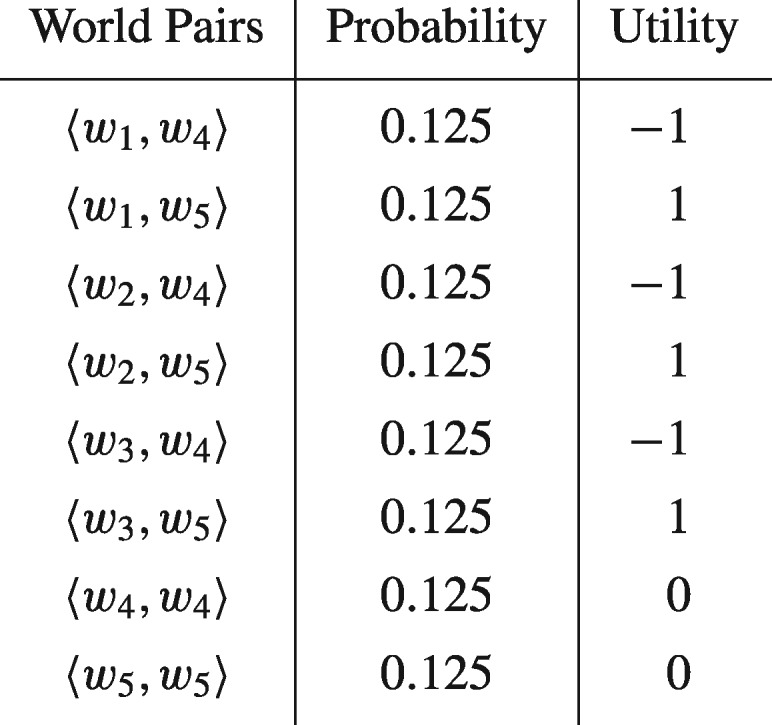


Let *Prob* and *Des* be pair of probability and desirability functions on ℘(W) constructed from *p* and *u* in the manner previously outlined by application of the standard axioms of probability and desirability. It is easy to see that the preferences induced by *Des* will violate preference actualism. In particular they will be such that:

(6) $$(\text{A}\cap \text{B},{\bar{\text{B}}}_{\bar{\text{A}}})≻(\text{A}\cap \text{B},{\text{B}}_{\bar{\text{A}}}),$$

(7) $$(\text{A}\cap \bar{\text{B}},{\text{B}}_{\bar{\text{A}}})≻(\text{A}\cap \text{B},{\bar{\text{B}}}_{\bar{\text{A}}}).$$

But by construction they satisfy the standard preference axioms of Jeffrey’s decision theory. So it follows that preferences violating preference actualism, although not representable as expected-utility maximizing, may nonetheless be desirability-maximizing.

### 4.2 Modelling Allais's and Diamond’s preferences

Strictly speaking, Equation 4 does not quite represent Diamond’s preference in full. Recall that Diamond’s preference consists in preferring a lottery (say a coin toss) that results in either Bob or Ann receiving a kidney (alternative *L*) to giving the kidney to Ann without using a fair lottery (alternative *L_A_*) and also to giving the kidney to Bob without using a fair lottery (alternative *L_B_*). This is how Diamond might evaluate the constant alternatives:

$$\begin{array}{l} Des(L_{A})=Des(\text{H}\cap \text{A},{\text{A}}_{T}),\\ Des(L_{B})=Des(\text{H}\cap \text{B},{\text{B}}_{T}). \end{array}$$

But since the lottery can turn out in more than one way, if he is to satisfy Jeffrey’s equation, Diamond must evaluate its desirability as a weighted sum of the ways in which it might turn out. For instance,

$$Des(L)=0.5Des(\text{H}\cap \text{B},{\text{A}}_{T})+0.5Des(\text{T}\cap \text{A},{\text{B}}_{H}),$$

assuming that he believes the coin to have an equal chance of coming up heads as tails when it is tossed.

There is thus a Jeffery desirability function representing Diamond’s preference as long as there is a function *Des* that simultaneously satisfies:

$$\begin{array}{c} Des(\text{H}\cap \text{B},{\text{B}}_{T})<0.5Des(\text{H}\cap \text{B},{\text{A}}_{T})+0.5Des(\text{T}\cap \text{A},{\text{B}}_{H}),\\ Des(\text{H}\cap \text{A},{\text{A}}_{T})<0.5Des(\text{H}\cap \text{B},{\text{A}}_{T})+0.5Des(\text{T}\cap \text{A},{\text{B}}_{H}). \end{array}$$

Since what motivates Diamond’s preference is his concern for fairness, he is (let us suppose) indifferent between Bob and Ann actually receiving the kidney. Moreover, the value generated by having used the lottery (or the disvalue generated by not having used the lottery) is, according to Diamond, independent of whether Ann or Bob actually receives the kidney. Hence, for Diamond,

0.5Des(H∩B,AT)+0.5Des(T∩A,BH)=Des(H∩B,AT)=Des(T∩A,BH)

$$Des(\text{H}\cap \text{B},{\text{B}}_{T})=Des(\text{H}\cap \text{A},{\text{A}}_{T}).$$

Therefore, to be able to represent Diamond’s preference as maximizing Jeffery desirability, all that is required is that there is a Jeffery desirability function such that

$$Des(\text{H}\cap \text{B},{\text{B}}_{T})<Des(\text{H}\cap \text{B},{\text{A}}_{T}).$$

That is, all we need is that there be a Jeffrey function that can represent a preference that violates preference actualism. In the last subsection we saw that such a function exists.

The same can be said for Allais's preference, namely, that it is only partly captured by Equation 5. But again, it is not hard to show that in Allais's case all that needs to be established is that there is a desirability function such that Des(R$\cap \bar{\text{G}}\cap \text{N}$, ${\text{G}}_{\text{\&}\bar{\text{R}}})<Des($R$\cap \bar{\text{G}}\cap$N, ${\bar{\text{G}}}_{\bar{\text{R}}})$. And this follows from what we established in the last subsection.

## 5 Ethical Actualism and Separability

We have argued that there are rational patterns of preference that are desirability-maximizing but not expected-utility maximizing. In this last section we turn to the question of what additional assumptions are needed for an agent’s preferences to be representable not just by a desirability function, but by a desirability function that takes the form of an expected utility. Our ambitions are three-fold: to establish the formal relationships between various salient properties of value functions, to exhibit the conditions that are necessary for expected-utility maximization, and to argue that these additional conditions are too strong to apply generally and hence that rationality does not require expected-utility maximization.

Let us begin by defining more carefully what it means for a desirability function to be an expected utility. Recall that acts are modelled in our framework by propositions of the form $\langle {\text{Y}}_{1}$, … ,${\text{Y}}_{n}\rangle$, where each Y*_i_* is the consequence of choosing the action in question in the event that S*^i^*. An expected-utility representation of a preference relation is characterized by a particular form that the desirability of such propositions take, namely, their desirabilities are probability weighted averages of the desirabilities of the Y*_i_*. More exactly:

Expected Utility: A desirability function, *Des*, defined on a suppositional algebra of propositions is an expected utility on this algebra if and only if for any partition of suppositions $S=\{S^{i}\}$,
  $$Des(\langle {\text{Y}}_{1},...,{\text{Y}}_{n}\rangle )=\sum_{i=1}^{n}Des({\text{Y}}_{i}|{\text{S}}^{i}).Prob({\text{S}}^{i}).$$

Hereafter, EU theory should be understood as the claim that rational preferences can be represented by a desirability function that is an expected utility as defined here. It should be noted, however, that this definition of an expected utility is somewhat more general than the usual one, in that it allows that the desirabilities of consequences be dependent on the states of the world in which they are realized. In the event that state-independence holds, it follows that $Des({\text{Y}}_{i}|{\text{S}}^{i})=Des({\text{Y}}_{i})$. Then if we let act *f* be the proposition $\langle {\text{Y}}_{1}$, … ,${\text{Y}}_{n}\rangle$ and $f({\text{S}}^{i})={\text{Y}}_{i}$, we obtain the familiar Savage formulation of expected utility: $Des(f)=\sum_{i=1}^{n}Des(f({\text{S}}^{i})).Prob({\text{S}}^{i})$.

Although state-dependence is natural in Jeffrey’s framework, only some versions of EU theory allow for it (for example, Karni [1985]). Accommodating state-dependence has the important and beneficial implication that the expected utilities of actions with coarse-grained consequences can be computed, so that we can dispense with the usual requirement of (for example, Savage’s) EU theory that consequences be maximally specific. But another problematic requirement of the theory—that the states of the world be probabilistically independent of the acts—cannot. For as we will show in Section 5.3, such independence is implied by the EU theory formulated here. But first we tackle our main objective, namely, showing that a preference relation that can be represented as maximizing desirability can also be represented as maximizing expected utility just in case it satisfies both a separability condition and a condition of ethical actualism.

### 5.1 Independence and additive separability

We have noted at various points that EU theory implies that the agent’s preferences are separable or that they can be represented by an additively separable utility function. Our next task is to make precise what this requirement amounts to in the framework in which we are working. Intuitively, two sets of propositions are separable from the point of view of some agent if their preferences for the members of one of the sets are independent of the truth or falsity of the members of the other set. If we consider not the preferences but the desirabilities that represent them, this translates into the requirement that the desirability of any member of one set is independent of the truth of any proposition in the other.

In this context, the sets of propositions that are relevant are the sets of counterfactuals under disjoint suppositions. And the form of separability that is required by EU theory can be rendered as the principle that the desirability that any *Y_i_* would be the case if *S^i^* were true is independent of what would be the case if any supposition inconsistent with *S^i^* were true. More formally, given a set of disjoint suppositions $\left\{S^{i}\right\}$ and a desirability *Des*, it must be the case that for any ${\text{Y}}_{i^{*}}$ that

$$Des({\text{Y}}_{i^{*}}|{⋂}_{i\neq i^{*}}{\text{Y}}_{i})=Des(\text{W},{\text{Y}}_{i^{*}}).$$

Then it follows from the definition of conditional desirability^14^ that

Des(〈Y1,...,Yn〉)=Des(Y1|Y2,...,Yn)+Des(Y2,...,Yn)=Des(Y1)+Des(Y2|Y3,...,Yn)+Des(Y3,...,Yn)=Des(Y1)+Des(Y2)+...=∑i=1nDes(Yi).

When a numerical representation of preference takes this form then it is said to be additive or additively separable. So we can conclude that a desirability measure is additively separable over the *S^i^* if and only if the counterfacts under any supposition are desirabilistically independent of those under any other supposition disjoint to it.

In the light of this we can state the separability condition required for expected utility as follows:

Counterfact Separability: If ${\left\{{\text{S}}^{i}\right\}}_{i=1}^{n}$ is a set of *n* disjoint suppositions, then

$$Des(\langle {\text{Y}}_{1},...,{\text{Y}}_{n}\rangle )=\sum_{i=1}^{n}Des({\text{Y}}_{i}).$$

Just how strong a condition this is can be brought out by noting that if a desirability function is additively separable then the corresponding probability function is multiplicative, that is, for any Yi≉ W,

Counterfact Independence: If ${\left\{{\text{S}}^{i}\right\}}_{i=1}^{n}$ is a set of *n* disjoint suppositions, then:
  $$Prob(\langle {\text{Y}}_{1},...,{\text{Y}}_{n}\rangle )=\prod_{i=1}^{n}Prob({\text{Y}}_{i}).$$

The claim that counterfact separability implies counterfact independence is proven in the Appendix as Theorem 9. But it can be intuitively explained by the fact that the counterfacts cannot be desirabilistically independent of each other unless knowing that one of the counterfacts holds is irrelevant to how likely the other counterfacts are to be true. Note that this implication still holds even if counterfact separability is restricted to just a particular class of propositions, such as those that are maximally specific with regard to all that the agent cares about.

Counterfact independence is not a plausible candidate for a general rationality constraint on belief and it is easy enough to find counter-examples to the claim that it is. Suppose I know that a prize is contained in one and only one of two boxes. I am about to pick one of the boxes but before opening it I am told that were I to open the other box I would win the prize. I can infer immediately that if I open the box I intended then I will not win the prize. So the counterfacts under the supposition that I open one box are not independent of those under the supposition that I open the other, in violation of counterfact independence The fact that EU theory requires counterfact independence (as we shall shortly show) therefore suggests that EU theory is not a correct theory of rationality.

Let’s conclude by introducing another independence condition on belief that will turn out to be important in our discussion of EU theory, namely the requirement that the facts be probabilistically independent from the strict counterfacts. More precisely:

**Fact-Counterfact Independence:** If X $\cap {\text{S}}^{i}=Ø$, then:
  $$Prob(\text{X},{\text{Y}}_{i})=Prob(\text{X}).Prob({\text{Y}}_{i})$$

The two independence conditions are closely related, but not equivalent. In the presence of centring, fact-counterfact independence does indeed imply counterfact independence but the latter only implies the former in the presence of a further condition:

**Supposition Independence:**Prob(S*^i^*,${\text{Y}}_{i})=Prob({\text{S}}^{i}).Prob({\text{Y}}_{i}).$

Supposition independence says that the probability that Y*_i_* is or would be the case on the supposition that S*^i^* is independent of whether S*^i^* is true or not. It is much more compelling than the other two independence conditions and, arguably, the characteristic property of evidential supposition. In this context, however, its main significance lies in the following claim, which we prove in the Appendix as Theorem 7.

Probability Equivalence Theorem: Assume centring. Then fact-counterfact independence is equivalent to the conjunction of supposition independence and counterfact independence

We will show in the section after the next that fact-counterfact independence is also a consequence of EU theory. But the principle is implausibly strong as a rationality constraint. Suppose again that I know that a prize is contained in one and only one of two boxes. Then if I pick one of them and discover that there is no prize in it, I can be sure that if I had picked the other box, then I would have got the prize. So what is the case—namely, that the prize is not in the box I picked—determines what would have been case had I picked the other one.

It seems clear then that counterfactual reasoning does not typically satisfy fact-counterfact independence nor does rationality require that it be satisfied. In fact, certain theories of rational decision-making assume that rational agents violate it. In game theory with imperfect information, for instance, which concerns rational strategic decision-making for agents who are uncertain about what moves other players have already made, it is standardly assumed that a rational strategy for figuring out whether a player, P, has made a particular move, M, is to ask oneself what would happen if P did not make that move. If it turns out that not making move M would lead to a bad outcome for P, then that might reasonably lead one to increase one’s credence in the proposition that P has made move M. Nonetheless, as we shall see, fact-counterfact independence is implied by EU theory as we reconstruct it within a propositional framework (but not by Jeffrey’s weaker theory). We take it that a good theory of practical rationality should, if possible, avoid such implausible epistemic implications. Moreover, it seems particularly problematic if a theory of rational individual decision-making contradicts an assumption that is standardly made in the theory of rational strategic decision-making. Hence, this result casts doubt on the claim that EU theory is our best theory of practical rationality.

### 5.2 Ethical actualism

An additive desirability function is not yet an expected utility. An expected utility is an additive desirability that satisfies a version of a principle common to many decision theories and that we have termed ethical actualism. The basic intuition behind this principle is that only the actual world matters, so that the desirability of combinations of facts and counterfacts should depend only on the desirability of the facts. In this section, we consider several formulations of this principle and clarify its relationship to separability.

One way of expressing ethical actualism more formally is as follows:

World Actualism: $u(\langle w,w_{1},...,w_{n}\rangle )=u(w)$

World actualism says that the desirability that *w* is the actual world and that the *w_i_* worlds would be the case if the *A_i_* were depends only on the desirability of *w*. In other words, once it has been established what world is the actual one, then it should be a matter of indifference what the counteractual worlds are. The applicability of world actualism rests on the possibility of giving a complete description of everything that matters. If we were able to do so, then any way in which the counterfacts mattered to us in the actual world could be registered in the description we give of that world. It is not that the counterfacts themselves must be written into the descriptions of worlds—this would lead to contradiction when the counterfacts specified in the description of a world differed from those in counteractual worlds—but that any way in which these counterfacts bear on our evaluation of the facts must be specified. For instance, suppose the desirability of dining at home is sensitive to how good a meal one would have had if one had dined out at the local restaurant: the fact that one would have had a better meal at the restaurant causes one to regret eating at home and the fact that one would have had a worse meal makes one appreciate the home-cooked meal all the more. Then these facts—the regret or the appreciation one experiences in the light of the counterfacts—must be built into the description of the actual world if World Actualism is to obtain.

The problem with the condition of world actualism is thus that it might hold for one specification of the possible worlds, but not for a model in which they are specified more coarsely. So we should not think of it as condition that applies to every model of counterfactual possibility, but rather as a methodological principle that requires contingencies to be sufficiently finely individuated for world actualism to hold within the model. This principle is one that many decision theorists seem to endorse. For instance, Broome ([1991]) recommends just such a strategy of fine individuation as a way of avoiding Allais's and Diamond’s putative counterexamples to the separability of rational preference. In a nutshell, his claim is that if there is some property of the outcomes of a decision that makes it rational to value an outcome differently depending on whether it has the property or not, then the outcomes should be individuated in accordance with that property.

Contrary to what appears to be the common view, however, imposing World Actualism on a model by appropriate individuation of prospects does not suffice to ensure the additive separability of desirabilities. As we have already seen, additive separability requires that counterfacts under mutually exclusive suppositions be probabilistically independent. But world actualism alone does not imply anything about the probabilistic relations between the counterfacts. So the question of whether rationality requires expected-utility maximization is not settled by the question of whether world actualism is or is not a reasonable condition.

A much stronger and partition-independent version of ethical actualism—the quantitative analogue of the condition we termed preference actualism—takes us much closer to what is required for desirabilities to be expected utilities. Let S be a set of suppositions and suppose that X $\cap {\text{S}}^{i}=Ø$.

Prospect Actualism: $Des(\text{X},{\text{Y}}_{i})=Des(\text{X})$

Prospect actualism says that the desirability that *X* is the case and that the *Y_i_* would be on the contrary-to-fact supposition that *S^i^* depends only on the desirability that *X*. Or to put it slightly differently, once it is given that *X* then it does not matter what would be the case under any supposition inconsistent with the truth of *X*.

Although prospect actualism expresses a similar idea to World Actualism, the relationship between them is quite complicated. Given centring, prospect actualism implies world actualism; but the converse is not true. In fact, prospect actualism only follows from world actualism in conjunction with the assumption that the facts are stochastically independent of the strict counterfacts, a condition we previously formalized as fact-counterfact independence. (This claim is proven in the Appendix as Theorem 11.)

Prospect actualism substantially constrains how we may value outcomes. Suppose, for instance, that you have to choose between two restaurants. You go to restaurant A and are served a very poor meal. An acquaintance goes to the other restaurant and reports that they were served a very good meal. Are things worse overall than they would have been if it had been the case that you would have been served a poor meal at the other restaurant as well? The issue is not whether your judgement concerning the meal at restaurant A can depend on what the meal at restaurant B would have been like—surely it should not—but whether the prospect of having a poor meal at restaurant A when you would have had a good one at restaurant B is a worse one than that of having the poor meal at restaurant A when you would also have had a poor one at restaurant B.

In this case, the issue boils down to whether the badness associated with the difference between what is the case and what might have been if some other course of action had been pursued is built into the description of the actual state of affairs. In other cases, the plausibility of prospect actualism depends on the information contained in the description of the counterfactual circumstances. Suppose, for instance, that the acquaintance in our example reports that standards of food hygiene were very poor at the other restaurant. You know they have the same owner, so you infer that standards will also be poor at the restaurant you chose. This affects your view about the desirability of your choice. In other words, the desirability of the prospect of going to restaurant A is not independent of the supposition that had you gone to restaurant B, you would have found food hygiene standards to be very poor. So prospect actualism will be violated whenever there are either probabilistic or desirabilistic dependencies between the facts and the strict counterfacts.

Although prospect actualism is a very strong condition, it alone is not sufficient to constrain desirabilities enough for them to be expected utilities. But jointly with the assumption that the facts are probabilistically independent of the counterfacts, prospect actualism does entail that desirabilities are expected utilities. More formally, as we prove in the Appendix as Theorem 21:

First Sufficiency Theorem: Assume centring. If *Des* is a desirability representation of a preference relation ≿ that satisfies fact-counterfact independence and prospect actualism, then *Des* is an expected-utility representation of ≿.

### 5.3 Expected utility, separability, and ethical actualism

We are now in a position to make precise our earlier claim that separability and ethical actualism are independent, necessary conditions for expected-utility maximization. Let’s take each aspect in turn. First, as we prove in the Appendix as Theorems 15 and 18, strong forms of both separability and ethical actualism are required for expected-utility maximization. More exactly:

Necessity Theorem: Assume centring. If *Des* is an expected-utility representation of the preference relation ≿, then *Des* satisfies counterfact separability, prospect actualism, fact-counterfact independence, and counterfact independence.

The necessity theorem is surprisingly strong and forcefully demonstrates just how much more demanding the requirement that agents maximize expected utility is than the requirement that they maximize desirability. We consider it highly implausible that failure to satisfy all four conditions entails irrationality on the part of an agent. Hence we are doubtful that rationality requires us to maximize expected utility.

Second, as we noted earlier on, ethical actualism and separability are based on different, though consistent, intuitions. The former expresses the idea that only what actually happens matters, while the latter expresses the idea that the desirability of orthogonal counterfacts are independent of each other’s truth. It is not difficult to see that the counterfacts can be separable even if ethical actualism is false. To see this again, consider the set of prospects displayed below and suppose you think that the counterfacts do matter. Specifically, suppose that were *E* not the case then you would prefer *BOB* rather than *ANN*, in violation of ethical actualism. So you prefer *L*_1_ to *L_A_* (in virtue of the former dominating the latter) even when you know that *E*. Nonetheless, you regard the outcomes under *E* and ¬E as separable because your preference for *BOB* over ANN were it the case that ¬E is not affected by whether *BOB* or ANN would be the case if *E*. Hence $L_{B}≻L_{2}$.


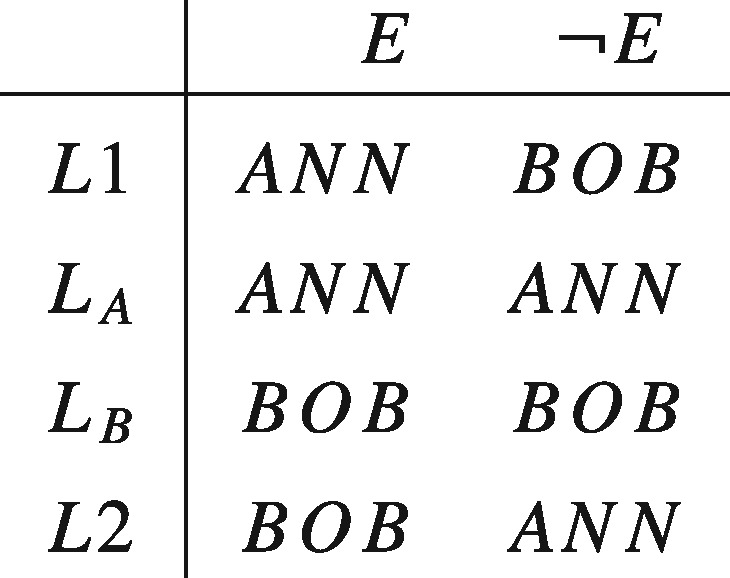


This example shows that satisfaction of ethical actualism is not necessary for separability. On the other hand, it might seem that ethical actualism should be sufficient for separability since if the counterfacts don’t matter, then trivially they will be desirabilistically independent of one another (they won’t matter whatever orthogonal counterfacts hold). But this intuition is false. Even if ethical actualism is true, the counterfacts can matter because they can be informative about what the facts are. If, for instance, I don’t know which box contains the prize, then I will—regardless of whether I am an actualist or not—care about whether it is true that if I were to open one of them, then I would find the prize, since learning this counterfact enables me to infer where the prize is.

What this example brings out is the possibility that the counterfacts matter because of probabilistic dependencies between facts and counterfacts. So one might hypothesize that when the counterfacts are probabilistically independent of the facts, then ethical actualism should imply separability. It turns out that this is true. More precisely, provided centring holds, prospect actualism and fact-counterfact independence jointly imply counterfact separability (we prove this in the Appendix as Theorem 13).

We have already observed that separability is not sufficient for ethical actualism. But prospect actualism, the strong form of ethical actualism required by EU theory, is a consequence of separability together with the following, weaker form of ethical actualism:

Restricted Actualism: $Des({\bar{\text{S}}}^{i},{\text{Y}}_{i})=Des({\bar{\text{S}}}^{i})$

Restricted actualism says that it does not matter that Y*_i_* would be the case under the supposition that S*_i_*, given that S*_i_* is false. Or to put it slightly differently, given that S*_i_* is not the case, it is a matter of indifference what would be the case if it were. Restricted actualism, like prospect actualism, is a partition-independent condition on evaluative attitudes, but it is quite a bit weaker than the latter. While prospect actualism clearly implies restricted actualism the latter only implies prospect actualism when the counterfacts are probabilistically and desirabilistically independent of each other. More formally, as we prove in the Appendix as Theorem 14, given centring, counterfact separability, and restricted actualism imply prospect actualism.

In virtue of the first sufficiency theorem, we can now infer a second set of sufficient conditions for a desirability function to be an expected utility, by drawing on the probabilistic equivalence theorem and the fact that counterfact separability implies Counterfact Independence. For then it follows, as we prove in the Appendix as Theorem 20:

Second Sufficiency Theorem: Assume centring. If *Des* is a Jeffrey representation of preference relation ≿ that satisfies counterfact separability, supposition independence, and restricted actualism, then *Des* is an expected-utility representation of ≿.

This second set of sufficient conditions is perhaps the more illuminating of the two since the dual dependence of EU theory on separability and ethical actualism is more transparent, as is the need for a distinct independence condition relating suppositions to beliefs about counterfacts under these suppositions. On the other hand, it somewhat obscures how demanding the probabilistic independence conditions are on expected-utility maximization. So, to finish, let us bring our various results together into a single statement relating EU theory and the two pairs of conditions on desirability and probability that have been discussed. It follows from the necessity theorem and the two sufficiency theorems that:

EU Equivalence Theorem: Let (*Des*, *Prob*) be a Jeffrey representation of a preference relation on a centred suppositional algebra. Then the following are equivalent:
  1. *Des* is an expected utility;
  2. *Des* satisfies prospect actualism and *Prob* satisfies fact-counterfact independence;
  3. *Des* satisfies both counterfact separability and restricted actualism and *Prob* satisfies supposition independence.

## 6 Concluding Remarks

We have seen that it is possible, when armed with an appropriate semantics, to extend Richard Jeffrey’s decision theory to counterfactual propositions. By doing so, one makes it possible to represent two preference patterns—those of Allais and Diamond—that have discomforted decision theorists for decades, and to rationalize them in terms of desirability maximization. We have also seen that when we add the conditions necessary for an expected-utility representation to this framework, we can no longer represent these intuitively rational preferences. Furthermore, the added postulates imply restrictions on the agent’s beliefs and desires that have little plausibility as rationality constraints. On the face of it, this seriously undermines EU theory’s claim to be the correct theory of practical rationality.

It might nonetheless be objected that this conclusion depends on the precise characterization of EU theory given in this article, and in particular on our partition-invariant formulation of it. This is only half-true. Restricting expected-utility maximization to prospects $\langle {\text{Y}}_{1}$, … ,${\text{Y}}_{n}\rangle$ such that the ${\text{Y}}_{i}$ are maximally specific will not invalidate our results, only restrict their scope. But this alternative characterization of EU theory still faces the following problem: It requires that maximally specific counterfacts under disjoint suppositions be both desirabilistically and probabilistically independent of each other and of the facts, which is not plausible as a requirement of rationality. It is true that it has already been recognized that Savage’s EU theory does not apply in circumstances in which the states of the world are not probabilistically independent of the acts. But granting this restriction still falls far short of recognizing that his theory does not apply whenever there are desirabilistic dependencies between the facts and the counterfacts. And to restrict application of expect utility theory to cases when there are no such dependencies would render it inapplicable in the circumstances imagined by Allais and Diamond. Either way, the claim that it provides a general theory of practical rationality cannot be sustained.

## Appendix

### A.1 Jeffrey representations

In this first section we present some useful results relating to Jeffrey representations of preferences on Boolean algebras. Let 〈Ω,⊆,W,Ø〉 be a complete, atomless Boolean algebra of propositions with upper bound W and lower bound Ø, and let ≿ be a preference relation on Ω. A pair of functions (*Des*, *Prob*) is a ‘Jeffrey representation’ of ≿ just in case *Prob* is a probability function on Ω and *Des* a desirability function on $\Omega^{\prime }=\Omega -\{Ø\}$ such that for all $\alpha ,\beta \in \Omega^{\prime },\;Des(\alpha )\geq Des(\beta )\Leftrightarrow \alpha ≿\beta$. Recall that a desirability function on $\Omega^{\prime }$ is a real-valued function such that for all $\alpha ,\beta \in \Omega^{\prime }$:

V1 (Normality): Des(W)=0
V2 (Desirability): If α∩β=Ø, then
  $$Des(\alpha \cup \beta )=\frac{Des(\alpha ).Prob(\alpha )+Des(\beta ).Prob(\beta )}{Prob(\alpha \cup \beta )}.$$

Recall also the definitions of conditional probability and desirability.

Conditional Probability: If Prob(α)≠0,then
  $$Prob(\beta |\alpha ):=\frac{Prob(\alpha \cap \beta )}{Prob(\alpha )}.$$

Conditional Desirability: If Prob(α∩β)≠0,then
  Des(β|α):=Des(α∩β)−Des(α).

**Lemma 2**

Let (Des, Prob) be a Jeffrey representation of ≿. Then:

1. $Des(\alpha ).Prob(\alpha )=-Des(\bar{\alpha }).Prob(\bar{\alpha })$
2. $\frac{Prob(\alpha )}{Prob(\bar{\alpha })}=-\frac{Des(\bar{\alpha })}{Des(\alpha )}$
3. If Des(α|β)=Des(α) and $Des(\bar{\alpha }|\beta )=Des(\alpha )$, then Prob(α|β)=Prob(α) and $Prob(\bar{\alpha }|\beta )=Prob(\alpha )$

**Proof**

Given that $\alpha \cup \bar{\alpha }=⊤$, it follows by the axioms of desirability and normality, that

$$Des(⊤)=Des(\alpha ).Prob(\alpha )+Des(\bar{\alpha }).Prob(\bar{\alpha })=0.$$

Hence $Des(\alpha ).Prob(\alpha )=-Des(\bar{\alpha }).Prob(\bar{\alpha })$. But if this is the case, then

$$\begin{array}{l} \Leftrightarrow Des(\alpha ).\frac{Prob(\alpha )}{Prob(\bar{\alpha })}=-Des(\bar{\alpha })\\ \Leftrightarrow \frac{Prob(\alpha )}{Prob(\bar{\alpha })}=-\frac{Des(\bar{\alpha })}{Des(\alpha )}. \end{array}$$

Assume that Des(α|β)=Des(α) and $Des(\bar{\alpha }|\beta )=Des(\bar{\alpha })$. Then by application of the above and from the fact that Des(·|β) is a desirability function,

Prob(α|β)Prob(α¯|β)=−Des(α¯|β)Des(α|β)=−Des(α¯)Des(α)=Prob(α)Prob(α¯).

Hence Prob(α|β)=Prob(α) and $Prob(\bar{\alpha }|\beta )=Prob(\bar{\alpha })$. ▪

### 7.2 Suppositional algebras

Hereafter our results pertain to suppositional algebras of propositions, where the latter are construed as sets of *n*-tuples of worlds. Let S=〈W,Ω,S,f,Ϝ,Γ〉 be a suppositional algebra with W a set of possible worlds, Ω& a Boolean algebra of subsets of W, $S=\{{\text{S}}^{i}\}\subseteq \Omega$ a set of *n* suppositions, *f* a selection function from W×S to Ω, Ϝ the set of *n*-tuples of worlds induced by *f*, and Γ a Boolean algebra of subsets of Ϝ (the set of all propositions). If *f* satisfies centring then we say that S is a centred suppositional algebra.

**Lemma 3**

*Assume that *S*is a centred suppositional algebra. Let X *⊆*S^i^. Then (X, *${\text{Y}}_{1}$*, … ,*${\text{Y}}_{n}$*) *=*(X *∩*Y_i_,*${⋂}_{j\neq i}{\text{Y}}_{j})$*.*

**Proof**

$$\text{(X, }{\text{Y}}_{1}\text{,...,}{\text{Y}}_{n})=\{\langle w_{0},w_{1},...,w_{n}\rangle :w_{0}\in \text{X and}w_{j}\in {\text{Y}}_{j}\}\text{. Since X }\subseteq S^{i}\text{, it follows from centring that }\langle w_{0},w_{1},...,w_{n}\rangle \in \text{ (X, }{\text{Y}}_{1}\text{,...,}{\text{Y}}_{n}\text{) }\Leftrightarrow w_{i}=w_{0}\text{. So:}$$

(X,Y1,...,Yn)={〈w0,w1,...,wn〉:w0∈X∩Yi and for all j,wj∈Yj}=(X∩Yi,Y1,...,Si,...,Yn)=(X∩Yi,⋂j≠iYj)

▪

#### 7.2.1 Probability conditions

In this section we prove a number of results concerning the relation between three different conditions of probabilistic independence. Throughout, let $S=\{S^{i}\}$ be a set of disjoint suppositions and ${\text{X}}_{i}\subseteq {\text{S}}^{i}$. Then consider:

Supposition Independence: $Prob({\text{S}}^{i},{\text{X}}_{i})=Prob({\text{S}}^{i}).Prob({\text{X}}_{i})$

Fact-Counterfact Independence: If X ∩ S${}^{j}=Ø$, then
  $$Prob(\text{X},{\text{Y}}_{j})=Prob(\text{X}).Prob({\text{Y}}_{j}).$$

Counterfact Independence: If ${\left\{{\text{S}}^{i}\right\}}_{i=1}^{n}$ is a set of *n* disjoint suppositions, then
  $$Prob(\langle {\text{Y}}_{1},...,{\text{Y}}_{n}\rangle )=\prod_{i=1}^{n}Prob({\text{Y}}_{i}).$$

**Theorem 4**

Fact-counterfact independence implies supposition independence.

**Proof**

Suppose that $\text{X}\subseteq {\text{S}}^{i}$. Then by fact-counterfact independence, since X $\cap {\bar{\text{S}}}^{i}=Ø$, it follows that

$$Prob({\bar{\text{S}}}^{i},{\text{X}}_{i})=Prob({\bar{\text{S}}}^{i}).Prob({\text{X}}_{i})$$

But then $Prob({\text{S}}^{i},{\text{X}}_{i})=Prob({\text{S}}^{i}).Prob({\text{X}}_{i})$.▪

**Theorem 5**

Let ${\text{X}}_{i}=X\cap S^{i}$ and assume centring. Then supposition independence implies that $Prob(X_{i})=Prob(X|S^{i})$*.*

**Proof**

Assume centring. Then,

$$Prob({\text{X}}_{i}|{\text{S}}^{i})=\frac{Prob({\text{S}}^{i},{\text{X}}_{i})}{Prob({\text{S}}^{i})}=\frac{Prob({\text{S}}^{i}\cap \text{X})}{Prob({\text{S}}^{i})}=Prob(\text{X}|{\text{S}}^{i}).$$

But Supposition Independence implies that $Prob({\text{X}}_{i}|{\text{S}}^{i})=Prob({\text{X}}_{i})$. Hence $Prob({\text{X}}_{i})=Prob(\text{X}|{\text{S}}^{i})$▪

**Theorem 6**

Let $S=\{S^{1},...,S^{n}\}$ be a set of n disjoint suppositions and suppose that for all S^i^,$S^{j}\in S,\;Prob(X_{i},Y_{j})=Prob(X_{i}).Prob(Y_{j})$. Then,

$$Prob(\langle {\text{Y}}_{1},...,{\text{Y}}_{n}\rangle )=\prod_{i=1}^{n}Prob({\text{Y}}_{i})$$

**Proof**

We prove the claim by induction on the number *n* of suppositions in S. By assumption the claim is true for *n* = 2, that is, that $Prob({\text{Y}}_{1},{\text{Y}}_{2})=Prob({\text{Y}}_{1}).Prob({\text{Y}}_{2})$. Assume true for *n* = *k*. Now,

Prob(Y1,...,Yk+1)=Prob(Y1,...,Yk|Yk+1).Prob(Yk+1)=Prob(Yk+1).∏i=1kProb(Yi|Yk+1)

in virtue of the induction hypothesis for *n* = *k* and the fact that $Prob(\cdot |{\text{Y}}_{\mathrm{k+1}})$ is a probability on the space of propositions. But by assumption, $Prob({\text{Y}}_{i},{\text{Y}}_{\mathrm{k+1}})=Prob({\text{Y}}_{i}).Prob({\text{Y}}_{i},{\text{Y}}_{\mathrm{k+1}})$. So $Prob({\text{Y}}_{1}$, … ,${\text{Y}}_{n})=\prod_{i=1}^{k+1}Prob({\text{Y}}_{i})$. ▪

**Theorem 7 (Probability Equivalence)**

Assume centring. Then Counterfact Independence and supposition independence are jointly equivalent to fact-counterfact independence.

**Proof**

Assume centring, Counterfact Independence and supposition independence. Suppose that S*^j^*= W − S*^i^*, X*_i_*$={\text{S}}^{i}\cap$ X = X and ${\text{Y}}_{j}={\text{S}}^{j}\cap$ Y = Y. It follows by centring and then Counterfact Independence that

Prob(X,Yj)=Prob(Si∩X,Yj)=Prob(Si,Xi,Yj)=Prob(Xi,Yj|Si).Prob(Si)=Prob(Xi|Si).Prob(Yj|Si).Prob(Si).

But by supposition independence,

$$Prob({\text{Y}}_{j}|{\text{S}}^{i})=Prob({\text{Y}}_{j}|\bar{{\text{S}}^{j}})=Prob({\text{Y}}_{j}).$$

Hence,

Prob(X,Yj)=Prob(Xi|Si).Prob(Yj).Prob(Si)=Prob(Si,Xi)Prob(Si).Prob(Yj).Prob(Si)=Prob(Si∩X).Prob(Yj)

in virtue of centring. So $Prob(\text{X},{\text{Y}}_{j})=Prob({\text{S}}^{i}\cap \text{X}).Prob({\text{Y}}_{j})$$=Prob(\text{X}).Prob({\text{Y}}_{j})$, in accordance with fact-counterfact independence.

Now assume fact-counterfact independence. Supposition independence follows by Theorem 4. Now for all S*^i^* and S*^j^* such that ${\text{S}}^{i}\cap$ S *^j^* = Ø,

$$Prob({\text{S}}^{i}\cup {\text{S}}^{j},{\text{X}}_{i},{\text{Y}}_{j})=Prob({\text{S}}^{i},{\text{X}}_{i},{\text{Y}}_{j})+Prob({\text{S}}^{j},{\text{X}}_{i},{\text{Y}}_{j}).$$

But by Lemma 3, centring implies that

$$\begin{array}{l} Prob({\text{S}}^{i},{\text{X}}_{i},{\text{Y}}_{j})=Prob({\text{S}}^{i}\cap \text{X},{\text{Y}}_{j}),\\ Prob({\text{S}}^{j},{\text{X}}_{i},{\text{Y}}_{j})=Prob({\text{S}}^{j}\cap \text{Y},{\text{X}}_{i}). \end{array}$$

And by fact-counterfact independence,

$$\begin{array}{l} \;Prob({\text{S}}^{i}\cap \text{X},{\text{Y}}_{j})=Prob({\text{S}}^{i}\cap \text{X}).Prob({\text{Y}}_{j}),\\ \;Prob({\text{S}}^{j}\cap \text{Y},{\text{X}}_{i})=Prob({\text{S}}^{j}\cap \text{Y}).Prob({\text{X}}_{i}),\\ Prob({\text{S}}^{i}\cup {\text{S}}^{j},{\text{X}}_{i},{\text{Y}}_{j})=Prob({\text{S}}^{i}\cup {\text{S}}^{j}).Prob({\text{X}}_{i},{\text{Y}}_{j}). \end{array}$$

So,

$$Prob({\text{X}}_{i},{\text{Y}}_{j})=\frac{Prob({\text{S}}^{i}\cap \text{X}).Prob({\text{Y}}_{j})+Prob({\text{S}}^{j}\cap \text{Y}).Prob({\text{X}}_{i})}{Prob({\text{S}}^{i}\cup {\text{S}}^{j})}.$$

But by Theorem 5, it follows from supposition independence that

$$\begin{array}{l} Prob({\text{Y}}_{j})=Prob(\text{Y}|{\text{S}}^{j}),\\ Prob({\text{X}}_{i})=Prob(\text{X}|{\text{S}}^{i}). \end{array}$$

So,

Prob(Xi,Yj)=Prob(X|Si).Prob(Y|Sj).Prob(Si)+Prob(Y|Sj).Prob(X|Si).Prob(Sj)Prob(Si∪Sj)=Prob(Y|Sj).Prob(X|Si)=Prob(Xi).Prob(Yj).

But by Theorem 6, if Prob(X*_i_*,${\text{Y}}_{j})=Prob({\text{X}}_{i}).Prob({\text{Y}}_{j})$ for all such X*_i_* and Y*_j_*, then $Prob({\text{Y}}_{1}$, … ,${\text{Y}}_{n})=\prod_{i=1}^{n}Prob({\text{Y}}_{i})$, in accordance with fact-counterfact independence. We conclude that, given centring, Counterfact Independence and supposition independence are jointly equivalent to fact-counterfact independence. ▪

**Corollary 8**

Let X $\cap Y_{i}=Ø$. Assume centring, Then fact-counterfact independence implies that

$$Prob(\text{X},{\text{Y}}_{1},...,{\text{Y}}_{n})=Prob(\text{X}).\prod_{i=1}^{n}Prob({\text{Y}}_{i}).$$

**Proof**

By the definition of conditional probability and fact-counterfact independence,

Prob(X,Yi,...,Yj)=Prob(X,Y1|Y2...,Yn).Prob(Y2...,Yn)

=Prob(X|Y2...,Yn).Prob(Y1|Y2...,Yn).Prob(Y2...,Yn)=Prob(X,Y2...,Yn).Prob(Y1)

by Theorem 6. Hence, by repeating the argument,

Prob(X,Yi,...,Yj)=Prob(X,Y2|Y3...,Yn).Prob(Y3...,Yn)=Prob(X|Y3...,Yn).Prob(Y2|Y3...,Yn).Prob(Y3...,Yn)=Prob(X,Y3...,Yn).Prob(Y1).Prob(Y2)...=Prob(X).∏i=1nProb(Yi)

▪

#### 7.2.2 Desirability-probability results

In this section we prove a number of results concerning the relation between three different conditions on desirabilities and the probabilistic independence conditions studied in the last section. As before, throughout let $S=\{S^{i}\}$ be a set of disjoint suppositions and ${\text{Y}}_{i}\subseteq$ S*^i^*. Then consider the following:

Restricted Actualism: $Des({\bar{\text{S}}}^{i},{\text{Y}}_{i})=Des({\bar{\text{S}}}^{i}).$
Prospect Actualism: If X $\cap {\text{S}}^{i}=Ø$, then
  $$Des(\text{X},{\text{Y}}_{i})=Des(\text{X}).$$

Counterfact Separability: If ∩ S*^i^*=Ø, then
  $$Des(\langle {\text{Y}}_{1},...,{\text{Y}}_{n}\rangle )=\sum_{i=1}^{n}Des({\text{Y}}_{i}).$$

**Theorem 9**

Counterfact Separability implies Counterfact Independence.

**Proof**

Let $S={\left\{{\text{S}}^{i}\right\}}_{i=1}^{n}$ be a set of *n* disjoint suppositions, S*^i^* any other supposition, and Y*_i_* any corresponding counterfactual proposition. We need to consider two cases separately. First let ${\text{Y}}_{j}$ be any proposition such that $Des({\text{Y}}_{j})\neq Des({\bar{Y}}_{j})$ (by the non-triviality assumption, such as ${\text{Y}}_{j}$ exists). Then by counterfact separability and the fact that (${\text{Y}}_{i}$,${\text{Y}}_{j})=\langle \text{W}$, (${\text{S}}^{1}{)}_{1},...,{\text{Y}}_{i}$,${\text{Y}}_{j},...,{\left({\text{S}}^{n}\right)}_{n})$:

$$\begin{array}{l} Des({\text{Y}}_{i},{\text{Y}}_{j})=Des({\text{Y}}_{i})+Des({\text{Y}}_{j})+\sum_{k\neq i,j}Des({\left({\text{S}}^{k}\right)}_{k}), \end{array}$$

$$\begin{array}{l} Des({\text{Y}}_{i},{\bar{\text{Y}}}_{j})=Des({\text{Y}}_{i})+Des({\bar{\text{Y}}}_{j})+\sum_{k\neq i,j}Des({\left({\text{S}}^{k}\right)}_{k}). \end{array}$$

But since (S*^k^*)*_k_* = Ϝ, it follows by normality that Des((S*^k^*)${}_{k})=0$. So $Des({\text{Y}}_{i}$, ${\text{Y}}_{j})=Des({\text{Y}}_{i})+Des({\text{Y}}_{j})$ and $Des({\text{Y}}_{i},{\bar{\text{Y}}}_{j})=Des({\text{Y}}_{i})+Des({\bar{\text{Y}}}_{j})$. But by the axiom of desirability:

Des(Yi)=Des(Yi,Yj).Prob(Yj|Yi)+Des(Yi,Y¯j).Prob(Y¯j|Yi)=[Des(Yi)+Des(Yj)].Prob(Yj|Yi)+[Des(Yi)+Des(Y¯j)].Prob(Y¯j|Yi)=Des(Yi)+Des(Yj).Prob(Yj|Yi)+Des(Y¯j).Prob(Y¯j|Yi).

But this can hold only if

$$Des({\text{Y}}_{j}).Prob({\text{Y}}_{j}|{\text{Y}}_{i})+Des({\bar{\text{Y}}}_{j}).Prob({\bar{\text{Y}}}_{j}|{\text{Y}}_{i})=0=Des({\text{Y}}_{j}).Prob({\text{Y}}_{j})+Des({\bar{\text{Y}}}_{j}).Prob({\bar{\text{Y}}}_{j})$$

by Lemma 2. By assumption $Des({\text{Y}}_{j})\neq Des({\bar{\text{Y}}}_{j})$. So $Prob({\text{Y}}_{j}|{\text{Y}}_{i})=Prob({\text{Y}}_{j})$ and hence $Prob({\text{Y}}_{i},{\text{Y}}_{j})=Prob({\text{Y}}_{i}).Prob({\text{Y}}_{j})$.

Now let ${\text{X}}_{j}$ be any proposition such that $Des({\text{X}}_{j})=Des({\bar{\text{X}}}_{j})$. Let ${\text{Y}}_{j}$ any proposition such that $Des({\text{Y}}_{j})\neq Des({\bar{\text{Y}}}_{j})$ and ${\text{X}}_{j}\cap {\text{Y}}_{j}=Ø$. Note that it follows from the axiom of desirability that $Des({\text{X}}_{j}\cup {\text{Y}}_{j})\geq \neq Des({\bar{\text{X}}}_{j}\cap {\bar{\text{Y}}}_{j})$. Then it follows from above that

$$\begin{array}{l} Prob({\text{Y}}_{i},{\text{X}}_{j}\cup {\text{Y}}_{j})=Prob({\text{Y}}_{i}).Prob({\text{X}}_{j}\cup {\text{Y}}_{j}),\\ \;Prob({\text{Y}}_{i},{\text{Y}}_{j})=Prob({\text{Y}}_{i}).Prob({\text{Y}}_{j}). \end{array}$$

But,

Prob(Yi,Xj∪Yj)=Prob(Yi,Xj)+Prob(Yi,Yj)=Prob(Yi,Xj)+Prob(Yi).Prob(Yj)Prob(Yi).Prob(Xj∪Yj)=Prob(Yi).Prob(Xj)+Prob(Yi).Prob(Yj).

It follows that $Prob({\text{Y}}_{i},{\text{X}}_{j})=Prob({\text{Y}}_{i}).Prob({\text{X}}_{j})$. Counterfact independence then follows from Theorem 6. ▪

**Theorem 10**

Assume centring. Then restricted actualism and supposition independence imply that $Des({\text{Y}}_{i})=[Des({\text{S}}^{i}\cap \text{Y})-Des({\text{S}}^{i})].Prob({\text{S}}^{i})$*.*

**Proof**

By the axiom of desirability,

Des(Yi)=Des(Si,Yi).Prob(Si|Yi)+Des(S¯i,Yi).Prob(S¯i|Yi)=Des(Si∩Y).Prob(Si|Yi)+Des(S¯i).Prob(S¯i|Yi)

in virtue of centring and restricted actualism. And by supposition independence, $Prob({\text{S}}^{i}|{\text{Y}}_{i})=Prob({\text{S}}^{i})=Prob({\bar{\text{S}}}^{i}|{\text{Y}}_{i})$. Hence

Des(Yi)=Des(Si∩Y).Prob(Si)+Des(S¯i).Prob(S¯i)=Des(Si∩Y).Prob(Si)−Des(Si)].Prob(Si)

by Lemma 2. Hence $Des({\text{Y}}_{i})=[Des({\text{S}}^{i}\cap \text{Y})-Des({\text{S}}^{i})].Prob({\text{S}}^{i})$. ▪

**Theorem 11**

Assume centring. Then world actualism and fact-counterfact independence imply prospect actualism.

**Proof**

Let $S={\left\{{\text{S}}^{i}\right\}}_{i=1}^{n}$ be a set of *n* disjoint suppositions and suppose that X $\subseteq {\text{S}}^{i^{*}}$. By centring, (X, Y_1_, … ,${\text{Y}}_{n})=(\text{X},({⋂}_{i\neq i^{*}}{\text{Y}}_{i})$) and by construction,

Des(X,Y1,...,Yn).Prob(X,Y1,...,Yn)=∑ωj∈(X,Y1,...,Yn)u(〈w0,w1,...,wn〉j).p(〈w0,w1,...,wn〉j)=∑ωju((w0)j).p(〈w0,w1,...,wn〉j)

by world actualism. But by centring and fact-counterfact independence, Prob(X, Y_1_, … , ${\text{Y}}_{n})=Prob(\text{X},{⋂}_{i\neq i^{*}}{\text{Y}}_{i})=Prob(\text{X}).Prob({⋂}_{i\neq i^{*}}{\text{Y}}_{i})$ and $p(\langle w_{0},w_{1},...,w_{n}\rangle )=p(w_{0}).p({⋂}_{i\neq i^{*}}w_{i})$. So,

∑ωju((w0)j).p(〈w0,w1,...,wn〉j)=∑w0∈Xu(w0).p(w0)[∑〈w1,...wn〉∈(Y1,...,Yn)p(⋂i≠i∗wi)]=∑w0∈Xu(w0).p(w0).Prob(⋂i≠i∗Yi).

Hence,

Des(X,Y1,...,Yn).Prob(X)=∑w0∈Xu(w0).p(w0)=Des(X).Prob(X).

It follows that Des(X,Y_1_, … ,${\text{Y}}_{n})=Des$(X) in accordance with prospect actualism. ▪

**Theorem 12**

Suppose that X $\cap (⋃{\text{S}}^{i}\in S)=Ø$. Then prospect actualism implies that Des(X,Y_1_, … ,${\text{Y}}_{n})=Des$(X).

**Proof**

By repeated applications of the definition of conditional desirability and prospect actualism,

Des(〈X,Y1,...,Yn〉)=Des(X,Y1|Y2,...,Yn)+Des(Y2,...,Yn)

=Des(X|Y2,...,Yn)+Des(Y2,...,Yn)=Des(X,Y2,...,Yn)=Des(X,Y2|Y3,...,Yn)+Des(Y3,...,Yn)...=Des(X,Yn)=Des(X)

▪

**Theorem 13**

Assume centring. Then fact-counterfact independence and prospect actualism imply counterfact separability.

**Proof**

By the axiom of desirability and then Lemma 11, given centring,

Des(〈Y1,...,Yn〉)=∑i=1nDes(Si,Y1,...,Yn).Prob(Si|〈Y1,...,Yn〉)=∑i=1nDes(Si∩Yi,⋂j≠i(Yj)).Prob(Si|〈Y1,...,Yn〉)=∑i=1nDes(Si∩Yi).Prob(Si|〈Y1,...,Yn〉)

in virtue of prospect actualism. Now by Corollary 8, given centring, fact-counterfact independence implies that

$$Prob({\text{S}}^{i}|\langle {\text{Y}}_{1},...,{\text{Y}}_{n}\rangle )=Prob({\text{S}}^{i}).$$

It follows that

Des(〈Y1,...,Yn〉)=∑i=1nDes(Si∩Yi).Prob(Si)=∑i=1nDes(Si∩Yi).Prob(Si)−∑i=1nDes(Si).Prob(Si)=∑i=1n[Des(Si∩Yi)−Des(Si)].Prob(Si),

in virtue of the fact that by V1 and V2, $\sum_{i=1}^{n}Des({\text{S}}^{i}).Prob({\text{S}}^{i})=0$. In particular,

Des(Yi)=Des(〈S1,...,Yi,...,Sn〉)=[Des(Si∩Yi)−Des(Si)].Prob(Si)+∑j≠i[Des(Sj)−Des(Sj)].Prob(Sj)

=[Des(Si∩Yi)−Des(Si)].Prob(Si).

Hence $Des(\langle {\text{Y}}_{1}$,...,${\text{Y}}_{n}\rangle )=\sum_{i=1}^{n}Des({\text{Y}}_{i})$. ▪

**Theorem 14**

Given centring, counterfact separability and restricted actualism imply prospect actualism.

**Proof**

Let ${\text{X}}_{i}=\text{X}\cap {\text{S}}^{i}$ and suppose ${\text{S}}^{j}=$ W − S*^i^*. Then by Lemma 3, given centring, Des(S*^i^*, ${\text{X}}_{i},{\text{Y}}_{j})=Des$(${\text{S}}^{i}\cap \text{X},\;{\text{Y}}_{j})$. But by the definition of conditional desirability and counterfact separability,

Des(Si,Xi,Yj)=Des(Xi,Yj|Si)+Des(Si)=Des(Xi|Si)+Des(Yj|Si)+Des(Si)=Des(Si,Xi)+Des(Si,Yj)−Des(Si)=Des(Si∩X)+Des(Si)−Des(Si)=Des(Si∩X),

in virtue of restricted actualism and centring. Hence $Des({\text{S}}^{i}\cap \text{X},{\text{Y}}_{j})=Des({\text{S}}^{i}\cap \text{X}$) in accordance with prospect actualism. ▪

### 7.3 Characterization results for expected utility

Throughout we assume that (Prob,Des) is Jeffrey representation of preferences defined on a centred suppositional algebra, Γ, of propositions. Let $S=\{{\text{S}}^{i}\}$ be a set of disjoint suppositions and ${\text{Y}}_{i}\subseteq$ S*^i^*. Recall that a desirability function, *Des*, defined on a centred suppositional algebra of propositions is an ‘expected utility’ on this algebra if and only if

$$Des(\langle {\text{Y}}_{1},...,{\text{Y}}_{n}\rangle )=\sum_{i=1}^{n}Des({\text{Y}}_{i}|{\text{S}}^{i}).Prob({\text{S}}^{i}).$$

#### 7.3.1 Necessity results

**Theorem 15**

Let *Des* be an expected utility. Then

1. $Des({\text{Y}}_{i})=[Des({\text{S}}^{i}\cap {\text{Y}}_{i})-Des({\text{S}}^{i})].Prob({\text{S}}^{i});$
2. $Des(\langle {\text{Y}}_{1}$, … ,${\text{Y}}_{n}\rangle )=\sum_{i=1}^{n}Des({\text{Y}}_{i}).$

**Proof**

By definition, if *Des* is an expected utility, then

$$Des(\langle {\text{Y}}_{1},...,{\text{Y}}_{n}\rangle )=\sum_{i=1}^{n}Des({\text{Y}}_{i}|{\text{S}}^{i}).Prob({\text{S}}^{i}).$$

So, in particular, since ${\text{Y}}_{i}=\langle {\text{S}}_{1}$, … ,${\text{Y}}_{i}$ … ,${\text{S}}_{n}\rangle =\langle {\text{Y}}_{i},\;{⋂}_{j\neq i}{\text{S}}^{j}\rangle$, it follows that

Des(Yi)=Des(Yi|Si).Prob(Si)+∑j≠iDes(Sj|Sj).Prob(Sj)=Des(Yi|Si).Prob(Si),

since $Des({\text{S}}^{j}|{\text{S}}^{j})=0$. But by the definition of conditional desirability,

$$Des({\text{Y}}_{i}|{\text{S}}^{i})=Des({\text{S}}^{i}\cap {\text{Y}}_{i})-Des({\text{S}}^{i}).$$

So $Des({\text{Y}}_{i})=[Des({\text{S}}^{i}\cap {\text{Y}}_{i})-Des({\text{S}}^{i})].Prob({\text{S}}^{i})$. But then

$$\sum_{i=1}^{n}Des({\text{Y}}_{i})=\sum_{i=1}^{n}Des({\text{Y}}_{i}|{\text{S}}^{i}).Prob({\text{S}}^{i})=Des(\langle {\text{Y}}_{1},...,{\text{Y}}_{n}\rangle ).$$

Hence,

$$Des(\langle {\text{Y}}_{1},...,{\text{Y}}_{n}\rangle )=\sum_{i=1}^{n}Des({\text{Y}}_{i}).$$

**Theorem 16**

Let *Des* be an expected utility. Then Prob satisfies Supposition Independence.

**Proof**

Let ${\text{X}}_{i}=$ S*^i^*∩ X. By the axioms of normality and desirability,

Prob(Xi)=Des(X¯i)Des(X¯i)−Des(Xi)=Des(Si∩X¯).Prob(Si)−Des(Si).Prob(Si)Des(Si∩X¯).Prob(Si)+Des(Si∩X).Prob(Si),

by Theorem 15(1) and in virtue of the fact that *Des* is an expected utility. But then by application of the axiom of desirability to $Des({\text{S}}^{i}).Prob({\text{S}}^{i})$,

Prob(Xi)=Des(Si∩X¯).Prob(Si)−Des(Si∩X).Prob(Si∩X)−Des(Si∩X¯).Prob(Si∩X¯)Prob(Si).[Des(Si∩X¯)+Des(Si∩X)]=Des(Si∩X¯).Prob(Si∩X)−Des(Si∩X).Prob(Si∩X)Prob(Si).[Des(Si∩X¯)+Des(Si∩X)]=Prob(Si∩X).[Des(Si∩X¯)+Des(Si∩X)]Prob(Si).[Des(Si∩X¯)+Des(Si∩X)]

=Prob(Si,Xi)Prob(Si).

Hence $Prob({\text{S}}^{i},{\text{X}}_{i})=Prob({\text{X}}_{i}).Prob({\text{S}}^{i})$ in accordance with supposition independence. ▪

**Corollary 17**

If *Des* is an expected utility then *Des* satisfies counterfact independence and fact-counterfact independence.

**Proof**

By Theorem 15, *Des* satisfies counterfact separability and by Theorem 9, counterfact separability implies Counterfact Independence. Similarly, by Theorem 16, *Des* satisfies supposition independence and by Theorem 10, Counterfact Independence and supposition independence are jointly equivalent to fact-counterfact independence. ▪

**Theorem 18**

Let *Des* be an expected utility. Then *Des* satisfies restricted actualism.

**Proof**

By Theorem 16, *Prob* satisfies supposition independence. So $Prob({\text{S}}^{i}|{\text{Y}}_{i})=Prob({\text{S}}^{i})$ and by the axiom of desirability,

Des(Yi)=Des(Si,Yi).Prob(Si|Yi)+Des(Si,Yi).Prob(Si|Yi)=Des(Si∩Yi).Prob(Si)+Des(Si,Yi).Prob(Si)

by Lemma 3, given centring. But by Theorem 15, $Des({\text{Y}}_{i})=(Des({\text{S}}^{i}\cap {\text{Y}}_{i})$$-Des({\text{S}}^{i})).Prob({\text{S}}^{i})$. Hence by Lemma 2, $Des({\text{Y}}_{i})=Des({\text{S}}^{i}\cap {\text{Y}}_{i}).Prob({\text{S}}^{i})$$+Des({\text{S}}^{i}).Prob({\text{S}}^{i})$. So, in accordance with restricted actualism,

$$Des({\bar{\text{S}}}^{i},{\text{Y}}_{i})=Des({\bar{\text{S}}}^{i}).$$

▪

**Corollary 19**

Let *Des* be an expected utility. Then Des satisfies prospect actualism.

**Proof**

By Theorem 15, *Des* satisfies counterfact separability and by Theorem 18, it satisfies restricted actualism. So, by Theorem 14, it satisfies prospect actualism.

#### 7.3.2 Sufficiency results

**Theorem 20**

Assume that *Des* satisfies counterfact separability and restricted actualism and that *Prob* satisfies supposition independence. Then *Des* is an expected utility.

**Proof**

Let Y_i_ = Y ∩ S^i^. By counterfact separability,

$$Des(\langle {\text{Y}}_{1},...,{\text{Y}}_{n}\rangle )=\sum_{i=1}^{n}Des({\text{Y}}_{i}).$$

But by Theorem 10, restricted actualism and supposition independence imply that $Des({\text{Y}}_{i})=[Des({\text{S}}^{i}\cap {\text{Y}}_{i})-Des({\text{S}}^{i})].Prob({\text{S}}^{i})$. Hence,

Des(〈Y1,...,Yn〉)=∑i=1n[Des(Si∩Yi)−Des(Si)].Prob(Si)=∑i=1nDes(Si∩Yi).Prob(Si)−∑i=1nDes(Si).Prob(Si)=∑i=1nDes(Si∩Yi).Prob(Si),

in virtue of the fact that by V1 and V2, $\sum_{i=1}^{n}Des({\text{S}}^{i}).Prob({\text{S}}^{i})=0$. But by the definition of conditional desirability,

$$Des({\text{Y}}_{i}|{\text{S}}^{i})=Des({\text{S}}^{i}\cap {\text{Y}}_{i})-Des({\text{S}}^{i}).$$

So,

$$Des(\langle {\text{Y}}_{1},...,{\text{Y}}_{n}\rangle )=\sum_{i=1}^{n}Des(\text{Y}|{\text{S}}^{i}).Prob({\text{S}}^{i}).$$

▪

**Theorem 21**

Assume that *Des* satisfies prospect actualism and that *Prob* satisfies fact-counterfact independence. Then *Des* is an expected utility.

**Proof**

Let Y*_i_* = Y ∩ S*^i^*. By the axiom of desirability and then Lemma 11,

$$Des(\langle {\text{Y}}_{1},...,{\text{Y}}_{n}\rangle )=\sum_{i=1}^{n}Des({\text{S}}^{i},{\text{Y}}_{1},...,{\text{Y}}_{n}).Prob({\text{S}}^{i}|\langle {\text{Y}}_{1},...,{\text{Y}}_{n}\rangle )=\sum_{i=1}^{n}Des({\text{S}}^{i}\cap \text{Y},{⋂}_{j\neq i}\left({\text{Y}}_{j}\right)).\frac{Prob({\text{S}}^{i}\cap {\text{Y}}_{i},{⋂}_{j\neq i}\left({\text{Y}}_{j}\right))}{Prob({\text{Y}}_{i},{⋂}_{j\neq i}\left({\text{Y}}_{j}\right))}.$$

Now by Theorem 7, fact-counterfact independence implies counterfact independence, which implies, by Theorem 6, that $Prob({\text{Y}}_{i}|{⋂}_{j\neq i}\left({\text{Y}}_{j}\right))=$$Prob({\text{Y}}_{i})$. Similarly, by Corollary 8, fact-counterfact independence implies that $Prob({\text{S}}^{i}\cap$Y*_i_*$\left|{⋂}_{j\neq i}\left({\text{Y}}_{j}\right))=Prob({\text{S}}^{i}\cap {\text{Y}}_{i}\right)$. Hence,

$$\frac{Prob({\text{S}}^{i}\cap {\text{Y}}_{i}|{⋂}_{j\neq i}\left({\text{Y}}_{j}\right))}{Prob({\text{Y}}_{i}|{⋂}_{j\neq i}\left({\text{Y}}_{j}\right))}=\frac{Prob({\text{S}}^{i}\cap {\text{Y}}_{i})}{Prob({\text{Y}}_{i})}=Prob({\text{S}}^{i}|{\text{Y}}_{i}).$$

Similarly by Theorem 12, prospect actualism implies that $Des({\text{S}}^{i}\cap \text{Y},{⋂}_{j\neq i}\left({\text{Y}}_{j}\right))=Des({\text{S}}^{i}\cap \text{Y})$. So,

$$Des(\langle {\text{Y}}_{1},...,{\text{Y}}_{n}\rangle )=\sum_{i=1}^{n}Des({\text{S}}^{i}\cap \text{Y}).Prob({\text{S}}^{i}|{\text{Y}}_{i}).$$

But by Theorem 4, fact-counterfact independence implies that $Prob({\text{S}}^{i}|{\text{Y}}_{i})=Prob({\text{S}}^{i})$. Hence,

$$Des(\langle {\text{Y}}_{1},...,{\text{Y}}_{n}\rangle )=\sum_{i=1}^{n}Des({\text{S}}^{i}\cap \text{Y}).Prob({\text{S}}^{i})▪$$

## Footnotes

^1^ We will throughout this article use period for multiplication.

^2^ Lara Buchak ([2013]) has recently suggested a solution to the Allais paradox that relies on a slightly different interpretation of Allais's preference than the one we suggest here. Whereas we interpret people that display this type of preference as being regret-averse, she interprets them as being risk-averse. And she introduces a risk function that, in addition to a utility and probability function, represents a person’s attitudes and argues that rational agents maximize risk-weighted expected utility. A limitation of Buchak’s account, we think, is that her theory cannot rationalize Diamond’s preference, since her risk-weighted expected-utility function is such that the expected benefit of a lottery can never exceed the benefits of each of its prizes. If we are right in thinking that Allais's and Diamond’s preferences are two instances of a general type of preference—namely, counterfactual-dependent preference—then it is an advantage of our theory over Buchak’s that we can solve the two paradoxes in the same way—namely, by introducing counterfactuals into the domain of Jeffrey’s decision theory.

^3^ The possibility of representing Allais's preference as maximizing desirability would probably not have impressed Jeffrey himself, who was satisfied with Savage’s view that Allais's preference reveals some sort of error of judgement (Savage [1972], pp. 102–3; Jeffrey [1990], p. 722).

^4^ Jeffrey’s theory does not however require that there be such maximally specific propositions or, to put it differently, that the Boolean algebra of prospects contains atoms. We work with them for expositional purposes.

^5^ See (Joyce [1999], Chapter 3.4) chapter 3.4 for a fuller discussion.

^6^ See (Joyce[1999] Theorem 4.1).

^7^ We assume that both outcomes, *ANN* and *BOB*, are desirabilistically independent of the random events E and ¬E (for example, coin comes up heads/tails) that determine the result of the lottery.

^8^ Broome makes his suggestion for resolving the problem within Savage’s framework but, as he notes, this leads to other problems, most notably to a tension with what he calls the rectangular field assumption. As Jeffrey’s theory makes no such assumption, the solution looks more promising in his framework.

^9^ The explanation is only partial since a full explanation would, in the case of the Diamond paradox, give a philosophical account of why counterfactuals can have moral value and, in the case of the Allais paradox, give a psychological account of why people care about what could have been. But such a discussion would go beyond the topic of this article.

^10^ The fact that a conditional is zero-tolerant does not necessarily mean that its antecedent is false. Hence, some want to call such conditionals subjunctive conditionals rather than counterfactuals. That name is, however, not necessarily any better, since zero-tolerant conditionals are not always expressed in the subjunctive mood. Hence, we will stick with the term ‘counterfactual’.

^11^ However, the simple version of the multidimensional model that we will work with entails the so-called conditional excluded middle (CEM)—according to which it is either the case that if A were true then B would be true, or if A were true then B would be false—which together with weak centring entails centring. A more general version of this model does not entail CEM.

^12^ Nothing of any substance depends on this zero-normalization, which is introduced for mathematical convenience alone.

^13^ As is evident from this definition, *Des* is not defined for propositions with zero probability. In fact, the Jeffrey-Bolker theorem (reproduced in Section 3.2) assumes that agents don’t have evaluative opinions of such propositions.

^14^ See the Appendix for a statement of its definition.
